# Biocompatible nucleus-targeted graphene quantum dots for selective killing of cancer cells via DNA damage

**DOI:** 10.1038/s42003-021-01713-1

**Published:** 2021-02-16

**Authors:** Lei Qi, Tonghe Pan, Liling Ou, Zhiqiang Ye, Chunlei Yu, Bijun Bao, Zixia Wu, Dayong Cao, Liming Dai

**Affiliations:** 1 State key Laboratory of Ophthalmology, Optometry and Visual Science, Institute of Advanced Materials for Nano-Bio Applications, School of Ophthalmology and Optometry, School of Biomedical Engineering, Wenzhou Medical University, 270 Xueyuanxi Road, Wenzhou, 325027 China; 2 Department of General Surgery, The First Hospital of Qiqihar, Affiliated Qiqihar Hospital, Southern Medical University, Qiqihar, 161005 China; 3 Australian Carbon Materials Centre (A-CMC), School of Chemical Engineering, University of New South Wales, Sydney, NSW 2052 Australia

**Keywords:** Cancer, Drug discovery

## Abstract

Graphene quantum dots (GQDs) are nano-sized graphene slices. With their small size, lamellar and aromatic-ring structure, GQDs tend to enter into the cell nucleus and interfere with DNA activity. Thus, GQD alone is expected to be an anticancer reagent. Herein, we developed GQDs that suppress the growth of tumor by selectively damaging the DNA of cancer cells. The amine-functionalized GQDs were modified with nucleus targeting TAT peptides (TAT-NGs) and further grafted with cancer-cell-targeting folic acid (FA) modified PEG via disulfide linkage (FAPEG-TNGs). The resulting FAPEG-TNGs exhibited good biocompatibility, nucleus uptake, and cancer cell targeting. They adsorb on DNA via the π–π and electrostatic interactions, which induce the DNA damage, the upregulation of the cell apoptosis related proteins, and the suppression of cancer cell growth, ultimately. This work presents a rational design of GQDs that induce the DNA damage to realize high therapeutic performance, leading to a distinct chemotherapy strategy for targeted tumor therapy.

## Abstract (web summary)

Qi et al. develop nucleus targeting graphene quantum dots (GQDs) by modifying amine-functionalised GQDs with nucleus targeting TAT peptides. The resulting functionalised GQDs exhibit good biocompatibility, nucleus uptake, and cancer cell targeting. They can suppress growth of cancer cells by selectively inducing DNA damage.

## Introduction

The cell nucleus is the center where DNA transcription occurs and forms the basis of life. During DNA duplication, the replicative helicase unwinds double-stranded DNA (dsDNA) to obtain the single-stranded DNA (ssDNA). A recent study revealed that the leading-strand and lagging-strand DNA polymerases within a single replisome function independently and stochastically, leading to the discordance between the leading-strand and lagging-strand DNA^1^. Thus, the ssDNA exposure time could be longer than traditionally thought. During this period, the nucleus is fragile and vulnerable to attack. Therefore, the cell nucleus is an important target for chemotherapy drugs (e.g., cisplatin and doxorubicin) to induce the apoptosis of tumor cells through the DNA damage inside the nucleus^2,3^. Due to the lack of specific tumor targeting and the presence of intracellular biological barriers, carrier-free drugs cannot be efficiently and specifically targeting inside the nucleus^4^. As such, various nucleus-targeting nanocarriers have been developed to improve anticancer therapy by increasing the concentration and targeting efficiency of the drugs within the nucleus^5–7^. However, a fundamental understanding of the interactions between these nucleus-targeting nanocarriers and the nucleus is indispensable for the design and development of new efficient nucleus-targeting anticancer drugs.

Nucleus-targeting gold nanoparticles (AuNPs) have been previously demonstrated to cause cytokinesis arrest, leading to apoptosis of the cancer cells^8^. Thus, AuNPs alone could act as an anticancer therapeutic agent in a new approach to chemotherapy. However, the potential clinical application of AuNPs as anticancer chemotherapy drugs has been hindered by the side reactions of AuNPs with biological fluids/biomolecules and intracellular structures^9^ as well as the difficulties of excretion after their in vivo administration^10^.

In addition to AuNPs, certain other nanomaterials, particularly graphene quantum dots (GQDs)^11^, have been demonstrated to interact specifically with DNA^12^. GQDs are naturally nucleus-targeting nanomaterials due to their unique structures and other physicochemical properties^13,14^. Indeed, the use of GQDs for nucleus targeting has been demonstrated to enhance the cell nucleus accumulation and DNA cleavage activity of anticancer drugs with good biocompatibility in vivo^15–19^. GQDs combined with Cu^2+^, Zn^2+^, or Ni^2+^ ions could cleave DNA^20,21^, cause DNA conformational transitions to induce the oligonucleotide i-Motif structure^22,23^, or interact with C-rich promoters, inhibiting the ABC transporters of multiple multidrug-resistant genes^24^. Furthermore, it was demonstrated that GQDs could accumulate in the cell nucleus to induce the oxidative damage of DNA and nucleus in the presence of fetal bovine serum (FBS) in vitro^25^. However, in these studies, GQDs still play an auxiliary role in enhancing the anti-tumor effect of chemotherapy drugs. In considering the interactions between GQDs and DNA, we therefore wondered if GQDs alone can be used as the in vivo anticancer reagents—a possibility that has yet been recognized.

**Fig. 1:**
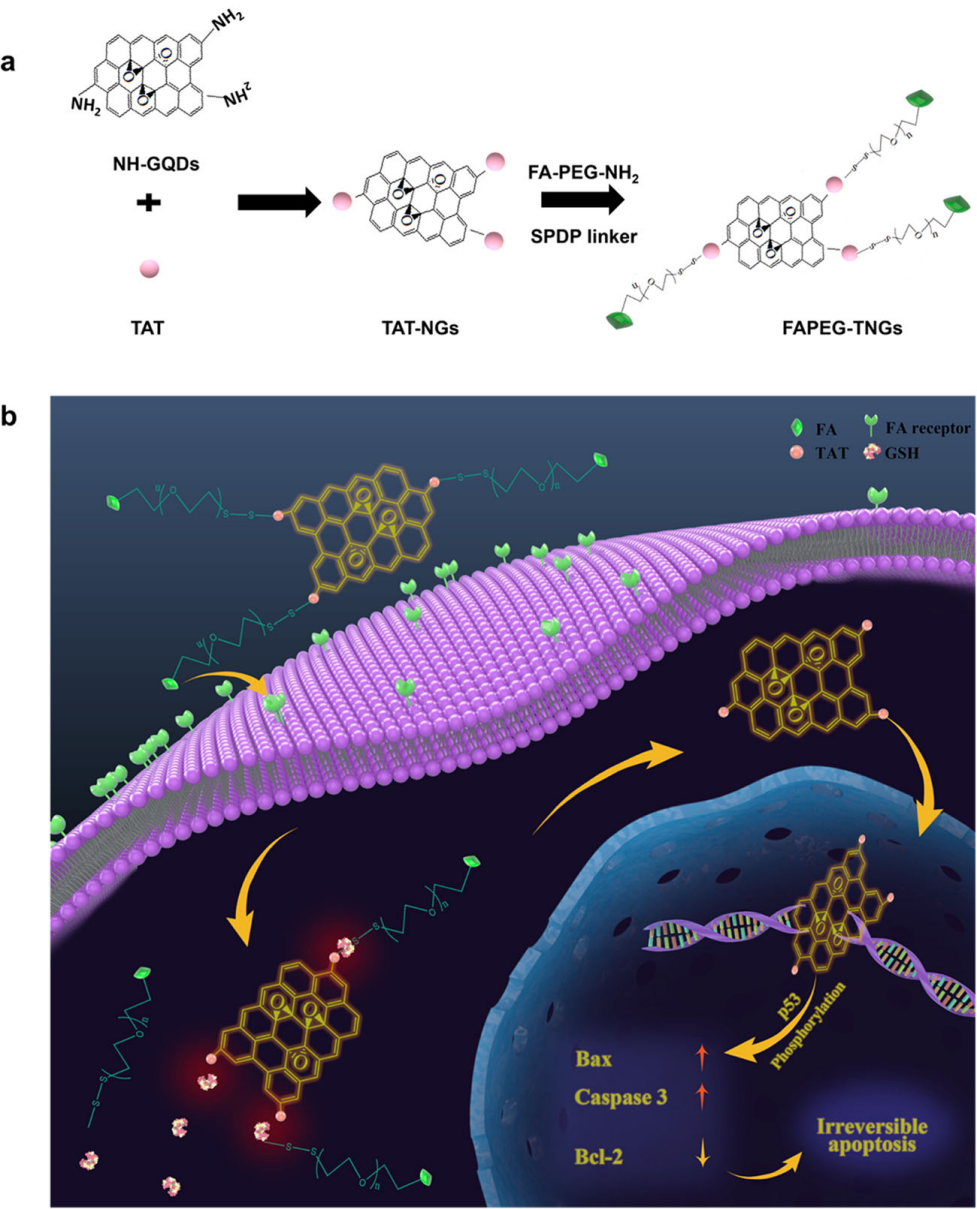
Schematic illustration of the FAPEG-TNGs preparation and the theraputic mechanism in cancer cell. **a** FAPEG-TNGs preparation. **b** the FAPEG-TNGs therapeutic mechanism in cancer cell.

By modifying GQDs with appropriate nucleus-targeting ligands in this study, we used GQD alone as an anticancer therapeutic reagent to directly damage the nucleus of cancer cells. As schematically shown in Fig. 1, we first prepared amine GQDs (NH-GQDs)^26,27^. Then, NH-GQDs were modified with the cell-penetrating and nucleus-targeting TAT peptides via amide formation (TAT-NGs), which can be recognized by importin *α* and *β* on the nuclear membrane^18^ for active transport from the cytoplasm into the cell nucleus^28,29^. The TAT-NGs were further modified with the cancer-cell-targeting FA-PEG (folic acid-modified polyethylene glycol) through S–S formation. The resultant FAPEG-TNGs with the targeting moieties can precisely target the folate receptor in the cancer cell membrane^30,31^ and prolong the circulation time of FAPEG-TNGs in the blood^32^. We observed good in vivo biocompatibility, cancer cell targeting, nuclear uptake, and enhanced anticancer effects of FAPEG-TNGs on *HeLa* tumor in vitro and in vivo. Furthermore, our extracellular study indicated that FAPEG-TNGs could adsorb on DNA rapidly and firmly through the π–π and electrostatic interactions, supporting the in vitro and in vivo observations. Our intracellular study found that FAPEG-TNGs induced DNA damage, which effectively activated the cell apoptosis-accelerating proteins to kill the cells consequently. In this study, therefore, we have rationally designed and developed cancer-cell-nucleus-targeting GQDs, and discovered that GQDs alone as an anticancer reagent can effectively and selectively kill cancer cells through DNA damage. This work represents a breakthrough in the development of chemotherapy strategies to use GQDs for targeted tumor therapy.

## Results

### Synthesis and structure of NH-GQDs, TAT-NGs, and FAPEG-TNGs

**Fig. 2:**
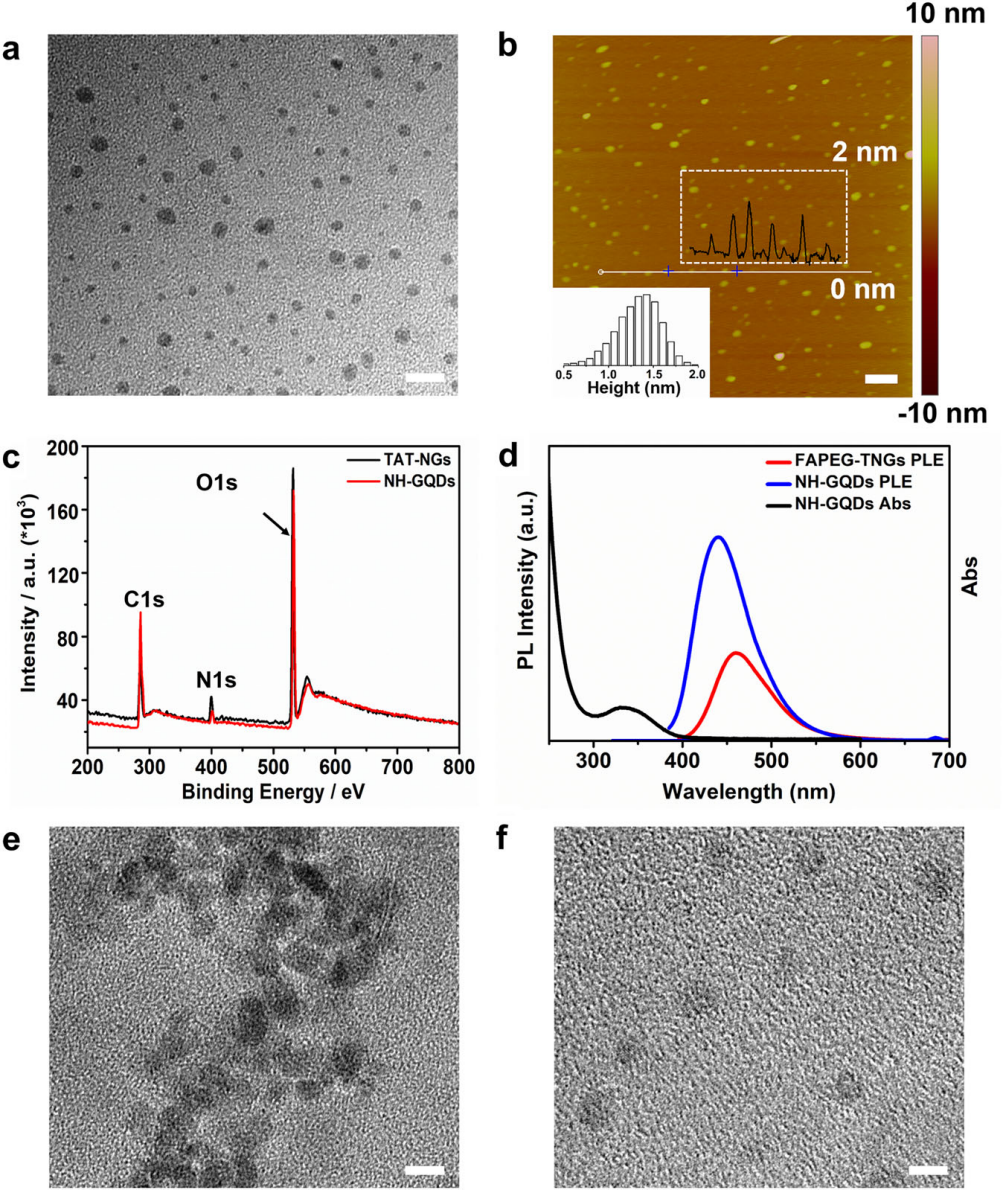
Characterization of the NH-GQDs derivatives. **a** HR-TEM image of NH-GQDs. **b** AFM image of NH-GQDs. **c** XPS full survey of NH-GQDs and TAT-NGs (10:1). **d** UV-Vis of NH-GQDs and *PL* spectra of NH-GQDs and FAPEG-TNGs. **e** HR-TEM images of TAT-NGs (10:1). **f** HR-TEM images of FAPEG-TNGs (scale bar of HR-TEM images, 10 nm; AFM image, 500 nm).

NH-GQDs were prepared through the hydrothermal method using citric acid and urea at 160 °C for 4 h^26,27^. To purify NH-GQDs, the dialysis tubes with three different cut-off molecular weights (500, 1000, and 3000 Da) were used. Determined by UV-Vis spectrum, the crude NH-GQDs showed one single peak at 336 nm (Supplementary Fig. 1). However, when dialyzed in 1000 or 3000 Da tubes, over 90% of crude NH-GQDs were removed, indicating that most of the NH-GQDs have molecular weights below 1000. Thus, the crude NH-GQDs were dialyzed in 500 Da tube and gained nearly 80%. As observed by high-resolution TEM (HR-TEM) images, the purified NH-GQDs are homogeneously distributed particles with an average diameter of approximately 5 nm (Fig. 2a and Supplementary Fig. 2a). Raman spectrum in Supplementary Fig. 2b shows a high graphitization degree with the ordered G band (I_G_ at 1589 cm^−1^) to disordered D band (*I*_D_ at 1348 cm^−1^) intensity ratio of 1.14. The corresponding atomic force microscopy (AFM) image in Fig. 2b indicates typical topographic heights in the range of 0.5–2 nm, with an average thickness of 1.5 ± 0.21 nm suggesting that most of the GQDs consist of 5–10 graphene layers^26,33^. The X-ray photoelectron spectroscopy (XPS) (Fig. 2c) of NH-GQDs shows the presence of O1s (50.37 at.%), N1s (4.07 at.%), and C1s (45.54 at.%) at 533, 400, and 284 eV, respectively. The high-resolution XPS C1s spectrum reveals the C = C (283.87 eV), C–N (285.27 eV), and C = O (288.0 eV) peaks (Supplementary Fig. 3a) while the high-resolution XPS N1s spectrum shows the C–N–C (398.76 eV) and N–H (400.51 eV) peaks (Supplementary Fig. 3b). The UV-Vis absorption spectrum of NH-GQDs shows one single peak at 336 nm attributable to the n–π* transition of C = O, while the corresponding *PL* spectrum displays a strong and stable peak at 440 nm with an optimal excitation wavelength of 340 nm (Fig. 2d).

Subsequently, NH-GQDs were modified by cell-nucleus-targeting TAT peptides with the reaction between the COOH-groups of the peptides and the NH_2_‑groups of NH-GQDs (Fig. 1). The amino terminus of the TAT peptide was acetylated to prevent self-connection of peptides. The mass ratio between TAT and NH-GQDs was set to 1:1, 5:1, and 10:1. The prepared TAT-NGs were purified by a centrifugal filter (3 kDa), which removes the free TAT peptides and NH-GQDs by molecular weight interception. The UV-Vis absorption spectrum of TAT-NGs has a single peak at 336 nm (Supplementary Fig. 4a). As determined by UV-Vis standard curve of NH-GQDs, the recovery rates were 44%, 64%, and 60% NH-GQDs for 1:1, 5:1, and 10:1, respectively. Fourier transform infrared (FT-IR) spectra (Supplementary Fig. 4b) show that the strong N–H stretch (3300 cm^−1^) peaks of NH-GQDs disappeared in the produced TAT-NGs, and was accompanied by the appearance of the carbonyl and amide bands at 1630 and 1380 cm^−1^. XPS spectra show a higher N content for TAT-NGs (10:1) than that of NH-GQDs (Fig. 2c), due to the additional N components from the TAT peptides. The XPS N1s spectrum reveals that the relative intensity of the N–H peak (401.72 eV) to the C–N–C peak (399.72 eV) for TAT-NGs decreased compared to that in NH-GQDs (Supplementary Fig. 3d), implying the formation of amide bonds to connect TAT peptides onto NH-GQDs. The zeta potential of NH-GQDs was −9.43 ± 0.3, while the zeta potentials of TAT-NGs (1:1, 5:1, 10:1) were determined to be −3.2 ± 0.03, 0.79 ± 0.2; 3.3 ± 0.4, respectively. This gradual change in the zeta potential with increasing TAT peptide ratio could reduce the repulsion of each TAT-NG nanoparticle and induce aggregation.

According to our cellular study, TAT-NGs (10:1) displayed a better ability for cell nucleus-targeting and stronger cell lethality with respect to TAT-NGs (1:1 and 5:1). Hence, TAT-NGs (10:1) were chosen mainly for subsequent study. The TAT-NGs (10:1) were grafted by FA-PEG (3400 Da, Fig. 1) by disulfide bond connection, which is expected not only to increase the dispersion^32^ and the blood circulation time for TAT-NGs, but also to target tumor cells by connecting them with the tumor-associated surface foliate receptor^30,32^. When FAPEG-TNGs enter cells, the disulfide bond can be cracked by glutathione (GSH) in the cells^34^ and release the TAT-NGs, which then enter the cell nucleus through TAT targeting induction (Fig. 1b). The by-product of the reaction was pyridine 2-thione (*P2*T) with a UV absorption peak at 343 nm (Supplementary Fig. 4c), indicating the successful execution of the reactions. The UV absorption of purified FAPEG-TNGs displays two peaks at 280 and 360 nm, characteristic of the FA moieties, which partially overlap with the absorption of NH-GQDs (Supplementary Fig. 4c). In addition, the maximum emission wavelength of FAPEG-TNGs is redshift to 460 nm relative to that of the NH-GQDs (Supplementary Fig. 2d) due to the influence of the grafted folic acid (Ex/Em: 370/455 nm). The FT-IR spectrum of FAPEG-TNGs shows the C–S and S–S bonds at 607 and 474 cm^−1^ (Supplementary Fig. 4d), confirming the S–S bond formation. As observed by HR-TEM image, the TAT-NG (10:1) nanoparticles were all aggregated by electrostatic interaction, but well dispersed after modification with FA-PEG (Fig. 2e, f). FA-PEG blocked the electrostatic interaction of TAT-NGs (10:1), allowing them to disperse well and to bind the tumor cells effectively. The observed average diameter of FAPEG-TNGs is approximately 8 nm. As PEG is difficult to be observed in TEM, this is actually the size of dispersed TAT-NGs.

### The targeting function of TAT-NGs and FAPEG-TNGs

#### Cell nucleus targeting

**Fig. 3:**
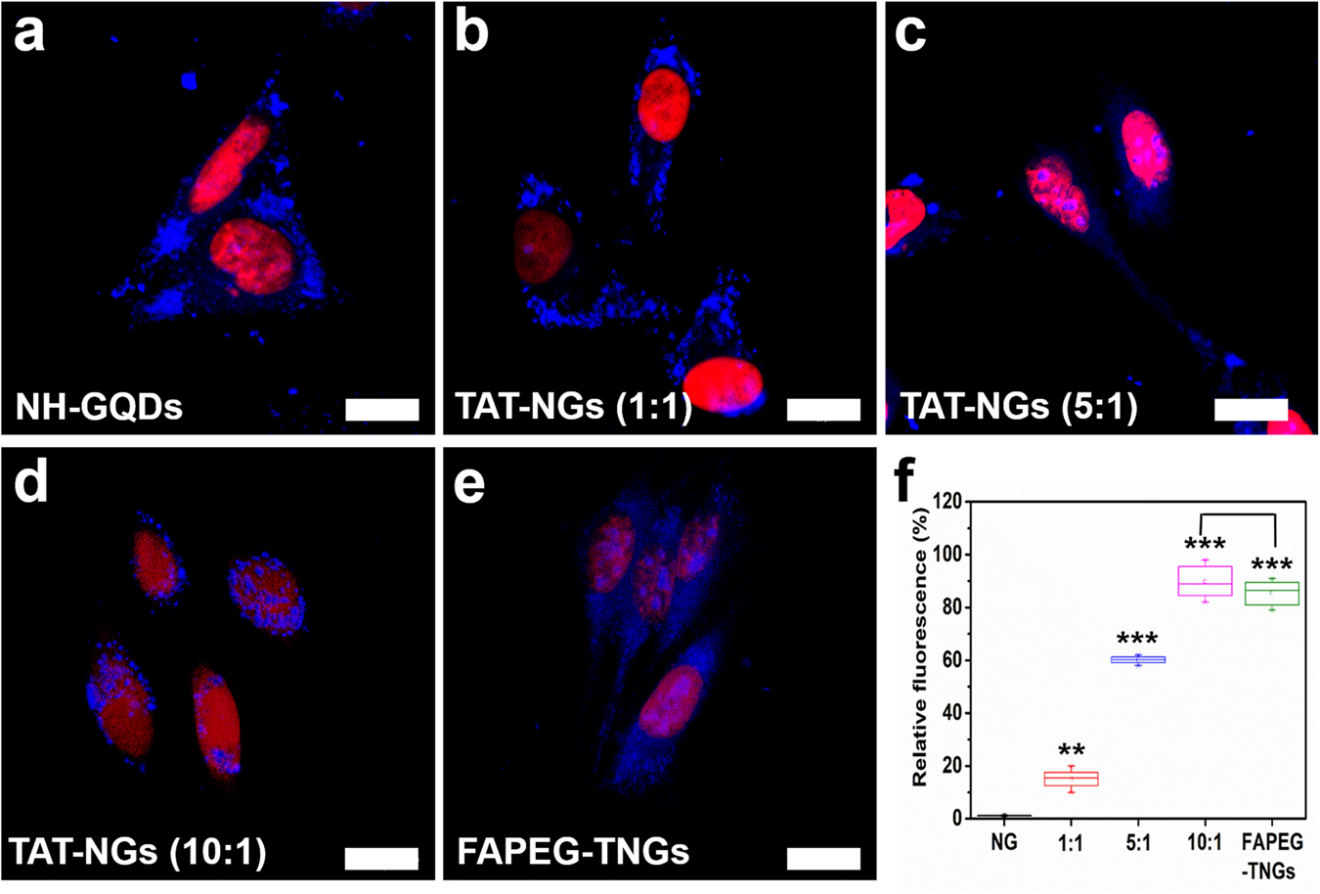
Fluorescence images of *HeLa* cells incubated with NH-GQDs derivatives. **a** NH-GQDs, **b** TAT-NGs (1:1), **c** TAT-NGs (5:1), **d** TAT-NGs (10:1), and **e** FAPEG-TNGs. **f** The endonuclear fluorescence intensity of TAT-NGs and FAPEG-TNGs relative to that of NH-GQDs (the images are overlaid with the blue color of NH-GQDs derivatives and the red color of nuclei stained with 7-aminoactinomycin D (7-AAD, Component B). Scale bars, 20 μm).

The cellular uptake and localization of NH-GQDs and TAT-NGs into cervical cancer *HeLa* cells were assayed by confocal laser scanning microscopy (CLSM). The cells were treated with NH-GQDs and TAT-NGs (1:1, 5:1, 10:1), separately. The intrinsic fluorescence of NH-GQDs was set as the control. After 24 h incubation, the NH-GQDs and TAT-NGs (1:1) were mostly located in the cytoplasm (Fig. 3a, b). However, TAT-NGs (5:1 and 10:1) were mostly observed in the cell nucleus, and nearly all TAT-NGs (10:1) were aggregated in the nucleus (Fig. 3c, d, Supplementary Fig. 5a, b, and Supplementary Videos 1 and 2). The endonuclear fluorescence intensity of the NH-GQDs and TAT-NGs was determined by ImageJ (Fig. 3f). The relative endonuclear fluorescence of TAT-NGs (5:1 and 10:1) was nearly 6- and 9-fold greater than that of NH-GQDs, implying that TAT peptide-modified NH-GQDs efficiently targeted and entered into the cell nucleus. Furthermore, when *HeLa* cells were treated with FAPEG-TNGs, most of the FAPEG-TNGs were observed in the cell nucleus (Fig. 3e, Supplementary Fig. 5c, and Supplementary Video 3). Their endonuclear fluorescence intensity was ~8-fold greater than that of NH-GQDs (Fig. 3f). The fluorescence in the cytoplasm was due to the released FA-PEG.

**Fig. 4:**
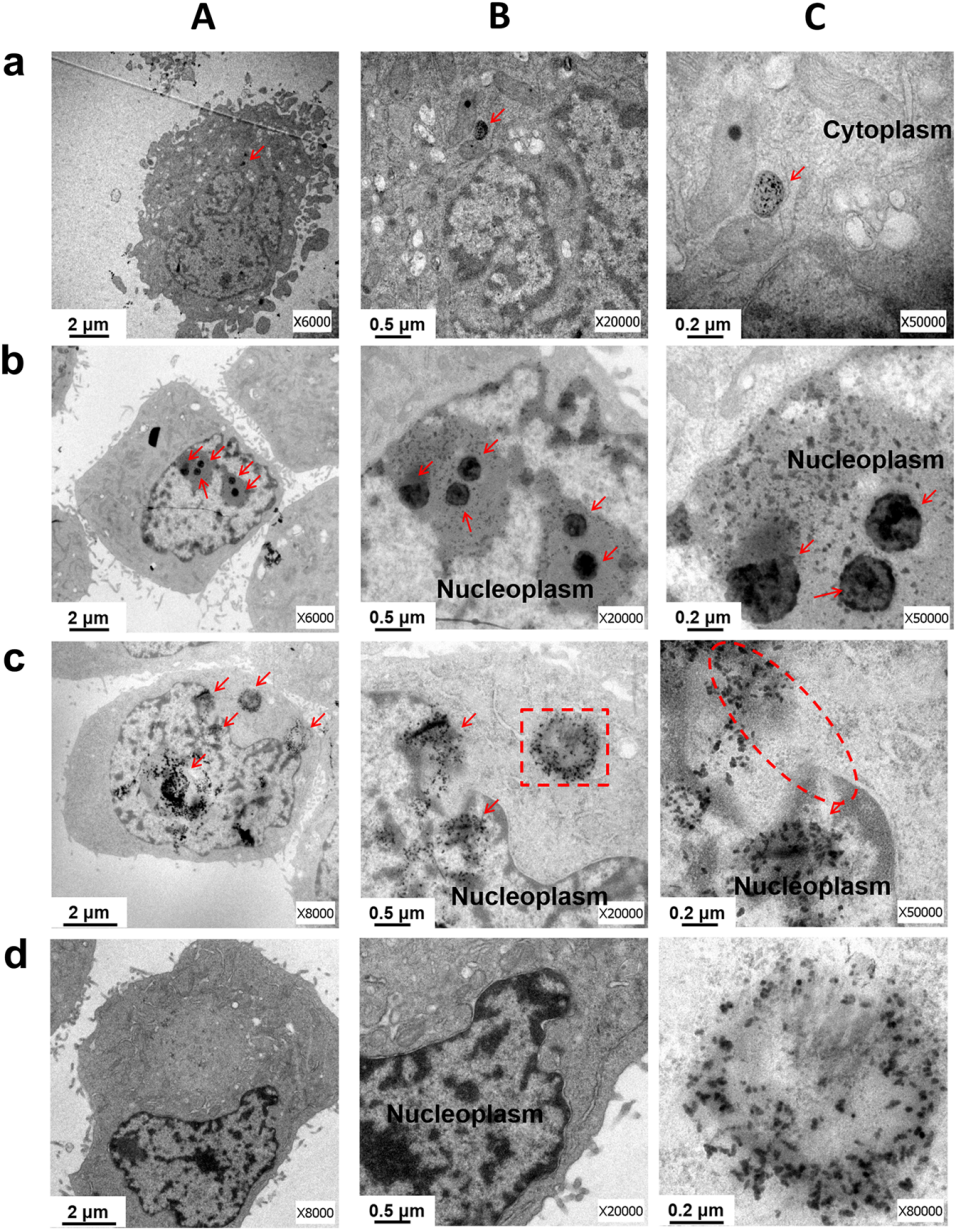
TEM images of *HeLa* cells treated with NH-GQDs derivatives. **a** NH-GQDs, **b** TAT-NGs (10:1), and (**c**) FAPEG-TNGs for 24 h (the images are zoomed in on the same cell from **A**, **B** to **C**; the red arrows show the NH-GQDs derivatives in the cells); **dA**, **dB** TEM images of *HeLa* cells without treatment; **dC** magnification of the square part in (**cB**) to observe the dots in the cells.

We further examined *HeLa* cell slices by TEM (Fig. 4) after treatment with NH-GQDs, TAT-NGs (10:1), or FAPEG-TNGs for 24 h. Comparing to the *HeLa* cells without treatment (Fig. 4dA, dB), we observed visible agglomerated dots in the NH-GQDs-, TAT-NGs (10:1)-, and FAPEG-TNGs-treated cells. These dots (Fig. 4cB marked with a square) were further magnified to show nano-sized particles characteristic of the GQDs derivatives (Fig. 4dC). Therefore, NH-GQDs, TAT-NGs (10:1), and FAPEG-TNGs all entered into cells. NH-GQDs were mainly located in the cytoplasm whereas both TAT-NGs (10:1) and FAPEG-TNGs crossed the nuclear membrane and located in the nucleoplasm (Fig. 4a–c). *HeLa* cells took up much more TAT-NGs (10:1) and FAPEG-TNGs than NH-GQDs. We also found that the cell nuclear membrane was obviously fractured in the FAPEG-TNGs-treated cells (Fig. 4cC marked with an ellipse). In the nucleoplasm, TAT-NGs (10:1) were agglomerated while FAPEG-TNGs were more dispersed. The TEM images reconfirm that TAT peptide-modified NH-GQDs were effectively targeted at the cell nucleus, and FA-PEG had little negative effects on the nucleus targeting.

#### Cancer cell-targeting by FAPEG-TNGs

**Fig. 5:**
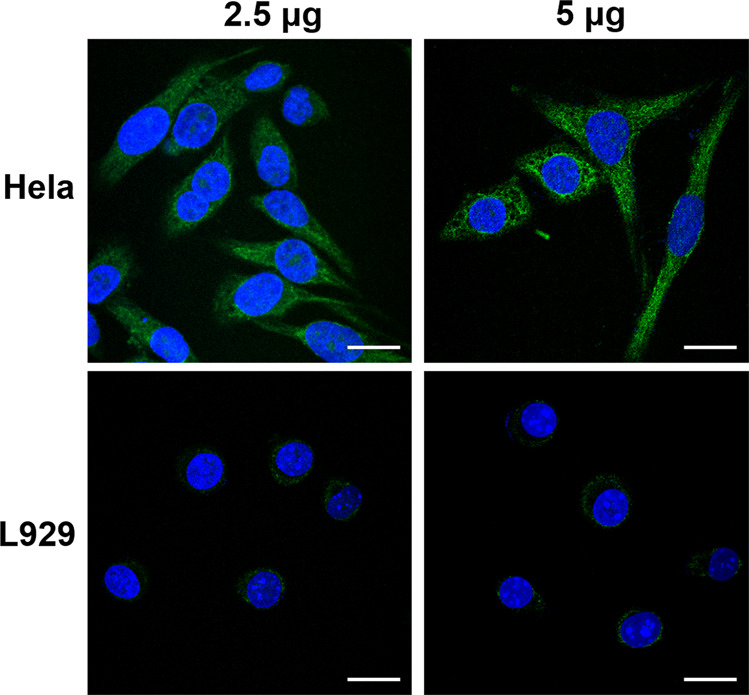
FR expression in *HeLa* and *L929* cells. Treated with 2.5 or 5 μg of anti-FOLR1 antibody, respectively. The images are overlaid with the green color of the FITC-tagged secondary antibody and the blue color of nuclei stained with Hoechst 33258. Scale bars, 20 μm.

As expected, FAPEG-TNGs targeted the tumor cells by binding to the overexpressed folic acid receptor (FR). Thus, the expression of FR in *HeLa* or the somatic cell lines of fibroblast *L929* cells was determined by immunofluorescence. After treated with anti-FOLR1 antibody, *HeLa* and *L929* cells were observed by CLSM (Fig. 5). *HeLa* cells expressed appreciable FR, but very little was expressed in *L929* cells. We doubled the quantity of anti-FOLR1 antibody, there was still very low green fluorescence in the *L929* cells. Therefore, FR was specifically overexpressed in the *HeLa* cells and FAPEG-TNGs preferentially targeted *HeLa* cells rather than *L929* cells.

### The cell lethality of TAT-NGs and FAPEG-TNGs

**Fig. 6:**
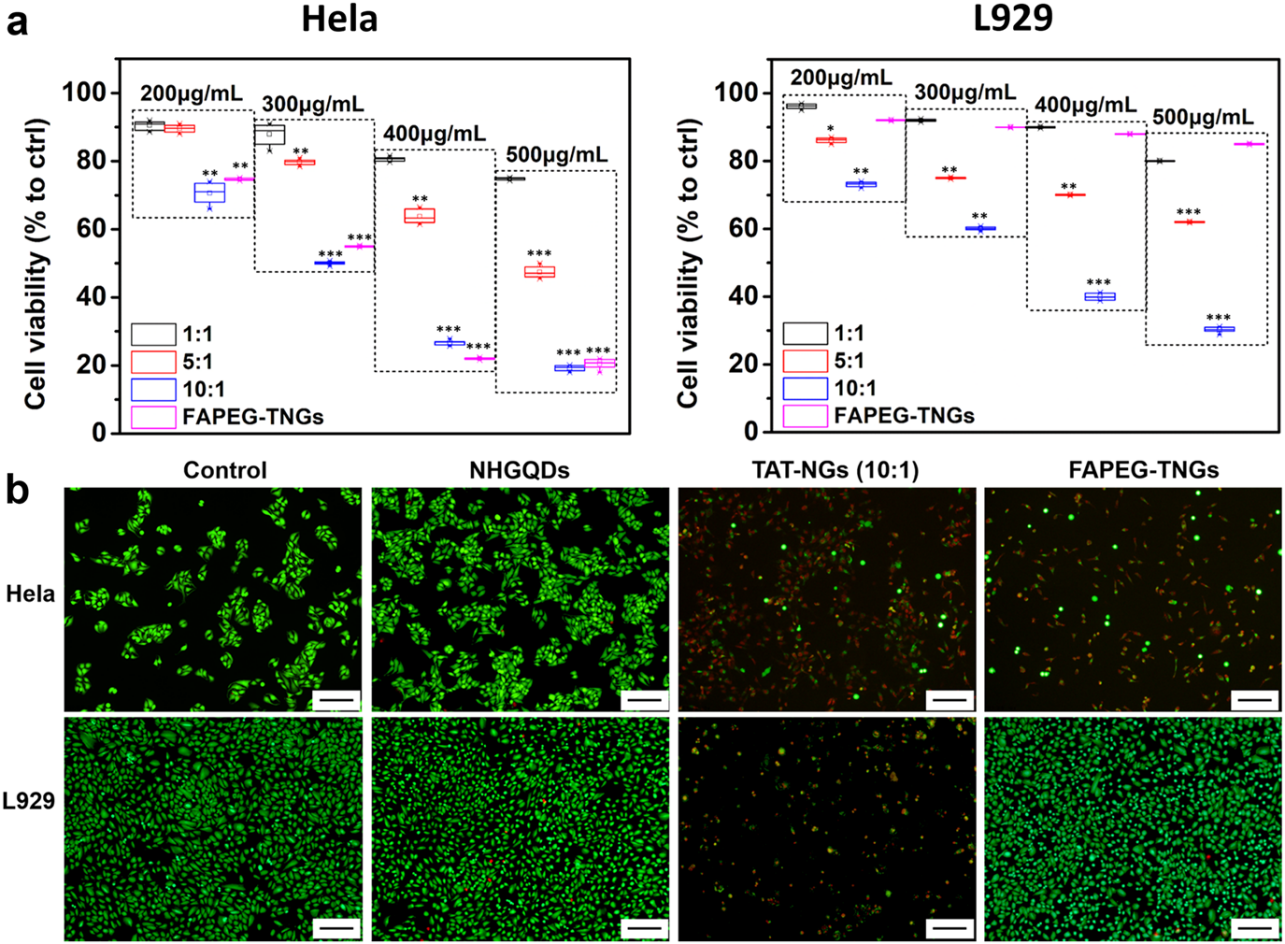
Cell viability of *HeLa* or *L929* cells treated with TAT-NGs and FAPEG-TNGs for 24 h. **a** CCK-8 assay. **b** Live/dead staining of cells by Calcein-AM/PI. Scale bars, 100 μm).

The influence of TAT-NGs (1:1, 5:1, and 10:1) and FAPEG-TNGs on cell proliferation was tested by CCK-8 assay using the cancer cell line of *HeLa* and the somatic cell line *L929*. After 24 h incubation, TAT-NGs (1:1) exhibited a low toxicity to both of *HeLa* and *L929* cells (Fig. 6a). However, TAT-NGs (5:1 and 10:1) had obvious lethality to the cells with no selectivity and the decrease in cell viability was dose-dependent. In particular, 80% of cells were killed by 500 μg mL^−1^ TAT-NGs (10:1) for 24 h treatment. FAPEG-TNGs exhibited a similar level of cell lethality towards *HeLa* cells. However, FAPEG-TNGs showed little toxicity to the somatic *L929*, indicating a good selectivity. To evaluate the long-term toxicity of FAPEG-TNGs to somatic cells, *L929* cells were treated with FAPEG-TNGs for 72 h (Supplementary Fig. 6). Compared to the 24-h exposure, the cell viability decreased, but there are still over 80% cells alive. This indicated, once again, the tumor cell targeting capability of FAPEG-TNGs. In addition, the cytocompatibility of NH-GQDs was evaluated in both *HeLa* and *L929* cells. Supplementary Fig. 7a, b shows that NH-GQDs had little toxicity to the cells even at 1000 μg mL^−1^, indicating good cytocompatibility of NH-GQDs. The cell lethality of TAT-NGs and FAPEG-TNGs was further evaluated on the tumor cell of human choroid melanoma cell line (*OCM-1*) and the somatic cell of human retinal pigment epithelial cell line (*ARPE-19*) (Supplementary Fig. 8). Similar to *HeLa* and *L929* cells, TAT-NGs (10:1) had significant lethality towards *OCM-1* cells and *L929* cells. Their cell viability decreased to lower than 20% when treated with TAT-NGs (10:1) at 500 μg mL^−1^. However, FAPEG-TNGs only had significant toxicity to *OCM-1* cells.

The lethality of FAPEG-TNGs was further investigated by the live/dead cell double-staining assay (Fig. 6b). In this case, *HeLa* or *L929* cells were incubated with 500 μg mL^−1^of NH-GQDs derivatives for 24 h. More than 90% of the *HeLa* cells died after the incubation with TAT-NGs (10:1) or FAPEG-TNGs, as shown by PI staining. However, *L929* cells were killed only by TAT-NGs (10:1). These results reconfirmed the tumor targeting capability of FAPEG-TNGs. In addition, most of the NH-GQDs-treated *HeLa* and *L929* cells were also green stained and alive, indicating that the NH-GQDs have good cytocompatibility.

### The extracellular influence of NH-GQDs derivatives on DNA

As discussed above, TAT-NGs (10:1) and FAPEG-TNGs entered the cell nucleus and induced severe cell lethality. Thus, the interaction between NH-GQDs derivatives and DNA is the key point to their anticancer mechanism. The ssDNA *P1* was synthesized and labeled by carboxyfluorescein-based dye (FAM, Ex: 480 nm) at the 3′ terminal^35^. NH-GQDs and the complementary sequence of *P1* (*HIV1*) showed no fluorescence emission (FLE) when excited at 480 nm (Supplementary Fig. 9).

**Fig. 7:**
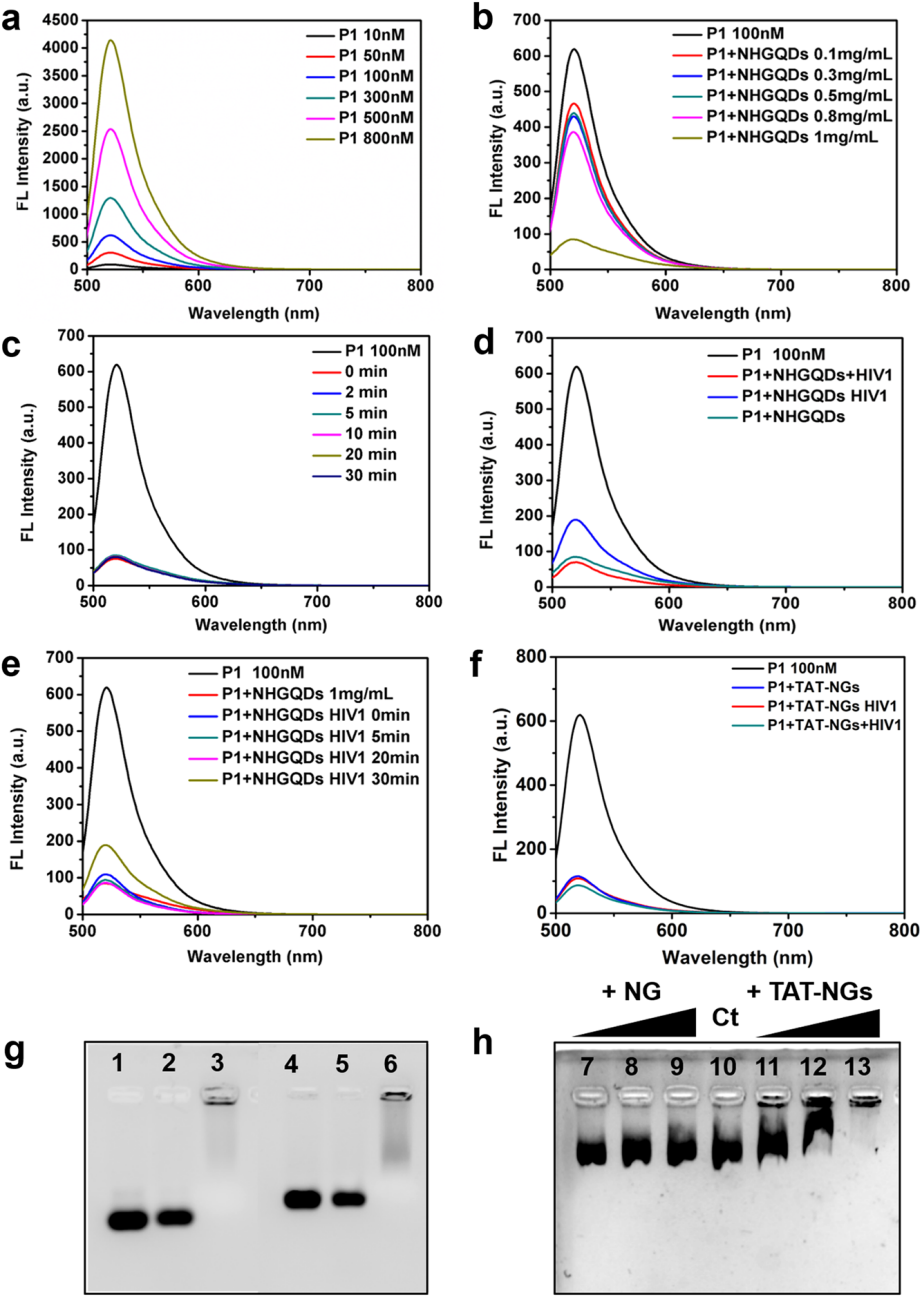
The interaction between NH-GQDs derivatives and DNA. **a** The FLE spectra of *P1* at different concentrations. **b**–**e** The FLE spectra of *P1* under different conditions. (**b)** *P1* mixed with different concentrations of NH-GQDs. (**c**) The fluorescence quenching of *P1* + NH-GQDs (1 mg mL^−1^) as a function of time. (**d**) The fluorescence intensity of ‘*P1* + NH-GQDs *HIV1*’ and *P1* + NH-GQDs+*HIV1*. (**e**) The fluorescence restoration of *P1* + NH-GQDs quenched by *HIV1* as a function of time. **f** The fluorescence intensity of *P1*, *P1* + TAT-NGs (10:1), *P1* + TAT-NGs (10:1) + *HIV1*, or ‘*P1* + TAT-NGs (10:1) *HIV1*’. **g** Gel electrophoresis diagram of *P2* (line 1) in addition with NH-GQDs (1 mg mL^−1^, line 2) or with TAT-NGs (10:1) (1 mg mL^−1^, line 3); gel electrophoresis diagram of *P3* (line 4) in addition with NH-GQDs (1 mg mL^−1^, line 5) or with TAT-NGs (10:1) (1 mg mL^−1^, line 6). **h** Gel electrophoresis diagram of *PL* (line 10) in addition with different concentrations of NH-GQDs (line 7: 1 μg, line 8: 2 μg, and line 9: 5 μg) or with different concentrations of TAT-NGs (10:1) (line 11: 1 μg, line 12: 2 μg, and line 13: 5 μg).

*P1* shows a strong concentration-dependent FLE peak at approximately 525 nm (Fig. 7a). Its FLE quenched in the presence of NH-GQDs (Fig. 7b). Particularly, ~90% fluorescence of *P1* quenched immediately when mixed with 1 mg mL^−1^ NH-GQDs (Fig. 7b, c). This indicates strong and fast adsorption of the ssDNA on NH-GQDs via π–π interactions (Supplementary Fig. 10a).

NH-GQDs, *P1*, and *HIV1* were mixed together for 30 min (‘*P1* + NH-GQDs+*HIV1*’ in Fig. 7d). Fluorescence was not recovered, indicating that NH-GQDs hindered the formation of dsDNA (Supplementary Fig. 10b). To verify this point, *P1* mixed NH-GQDs for 5 min, following the addition of *HIV1* (in blue, ‘*P1* + NH-GQDs *HIV1*’ in Fig. 7d), which reduced the adsorption of *HIV1* on the NH-GQDs. The fluorescence of ‘*P1* + NH-GQDs *HIV1*’ was partially recovered after 30 min of treatment (Fig. 7e), indicating the formation of dsDNA subsequently escape from the NH-GQDs. However, some residual ssDNA remained adsorbed on NH-GQDs. Therefore, the release of the adsorbed ssDNA from NH-GQDs was relatively slow (Supplementary Fig. 10c).

Similar to NH-GQDs, TAT-NGs (10:1) were found to strongly adsorb the *P1* upon mixing (Fig. 7f). When we mixed TAT-NGs, *P1,* and *HIV1* together (*P1* + TAT-NGs+*HIV1*) or added *HIV1* to the TAT-NGs and *P1* mixture (*P1* + TAT-NGs *HIV1*), however, neither of the two mixture solutions recovered its fluorescence. Thus, TAT-NGs might have adsorbed both ssDNA and dsDNA or adsorbed ssDNA more strongly than NH-GQDs do. When TAT-NGs enter cell nucleus, therefore, they could adsorb onto the genomic DNA and hinder the formation of dsDNA in the nucleus to induce nuclear damage.

In addition, the electrostatic interactions between NH-GQDs derivatives and DNA strands were examined by agarose gel electrophoresis, in which the DNA molecules display a negative charge and move to the positive electrode in the electric field. The ssDNA molecules with different sequence lengths were synthesized and named *P2* (47 bp) and *P3* (50 bp). A slight change was observed when *P2* or *P3* was incubated with NH-GQDs (Fig. 7g line 2 and line 5, respectively). In contrast, ssDNA molecules were stopped at the gel origin upon the incubation with TAT-NGs (10:1) (Fig. 7g line 3 and line 6, respectively). The zeta potential values of NH-GQDs and TAT-NGs (10:1) were −9.43 ± 0.3 and 3.3 ± 0.4, respectively. The positively charged TAT-NGs (10:1) altered the charge of ssDNA, halting its migration in the gel. Thus, the binding between TAT-NGs (10:1) and ssDNA was stronger than that between NH-GQDs and ssDNA due to the electrostatic adsorption.

We further investigated the interactions between NH-GQDs derivatives and supercoiled DNA (*PL*, 8 Kb). Similar to ssDNA, NH-GQDs did not change the movement of *PL* in the gel (Fig. 7h lines 7–9). However, *PL* migration was stopped by TAT-NGs (10:1) and the retention was gradually enhanced with increase in the TAT-NG (10:1) concentration (Fig. 7h lines 11–13). Clearly, TAT-NGs (10:1) displayed strong electrostatic adsorption of both ssDNA and supercoiled DNA.

### Induction of DNA damage in cancer cells by FAPEG-TNGs

#### Comet assay

**Fig. 8:**
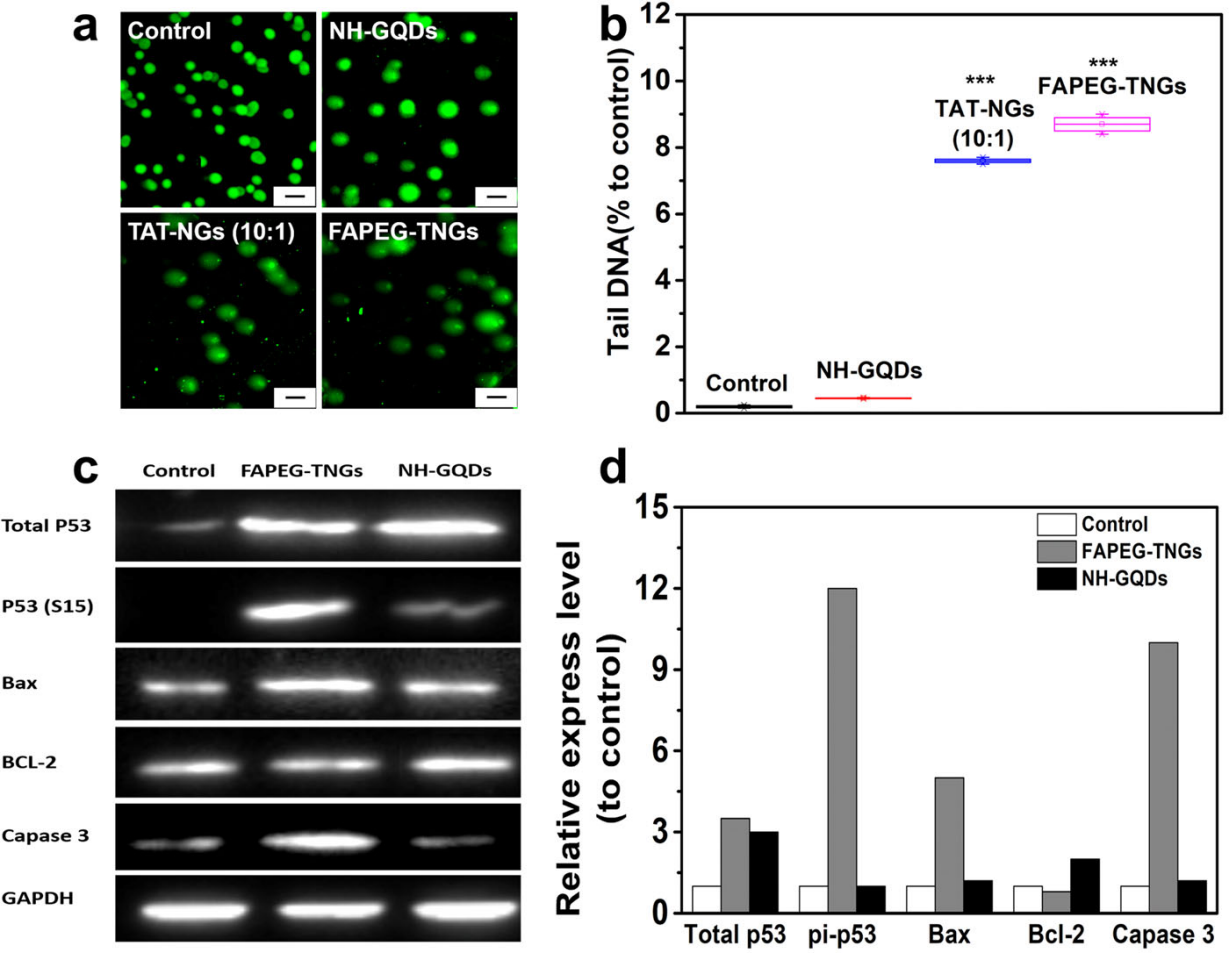
Induction of DNA damage in HeLa cells by NH-GQDs derivatives. **a** Example images of comet tails for *HeLa* cells treated with NH-GQDs, TAT-NGs (10:1), or FAPEG-TNGs for 24 h. Scale bars, 20 μm. **b** Statistics of the percentages of DNA in the tails of the cells, calculated using CASP software. **c** Western blot analysis of total p53, pi-p53, Bax, Bcl-2, Caspase 3, and GAPDH (reference protein) in *HeLa* cells after 24 h of treatment with FAPEG-TNGs or NH-GQDs. **d** Relative quantities of total p53, pi-p53, Bax, Bcl-2 and Caspase 3.

As discussed above, TAT-NGs entered into the cell nucleus and induced severe cell death. However, what happened inside the cells? To gain a mechanistic understanding, we used the Comet assay to determine the intracellular DNA damage of *HeLa* cells incubated with NH-GQDs derivatives for 24 h. The DNA chains of NH-GQDs-treated cells were spherical, indicating little DNA damage (Fig. 8a). However, DNA trailing were observed in the TAT-NGs (10:1)-treated and FAPEG-TNGs-treated cells, that were about ~8- and 9-fold higher in the tails of the control cells, respectively (Fig. 8b), implying that TAT-NGs (10:1) and FAPEG-TNGs damaged the DNA strands in *HeLa* cells. This implies that TAT-NGs (10:1) and FAPEG-TNGs entered the cell nucleus and induced the DNA damage, leading to the cell death.

#### Western blot (WB) analysis

To obtain the molecular mechanism underlying FAPEG-TNGs-induced DNA damage, the expression profiles of DNA damage and apoptosis-related genes were analyzed by WB in *HeLa* cells treated with NH-GQDs or FAPEG-TNGs for 24 h. The expression level of p53 protein was monitored, which would be activated by phosphorylation during DNA damage^36–38^. The total expression p53 was upregulated in both the FAPEG-TNGs-treated and NH-GQDs-treated cells (Fig. 8c). However, the phosphorylated p53 (pi-p53) was overexpressed only in the FAPEG-TNGs-treated cells and was 12-fold higher than in the untreated cells. Therefore, FAPEG-TNGs induced the DNA damage in *HeLa* cells. The pi-p53 acts as the tumor suppressor in many tumor types by inducing the apoptosis^39^. Thus, the apoptosis accelerator Bax and the apoptosis repressor Bcl-2 were further monitored by WB. The expression of Bax was upregulated by pi-p53 in the FAPEG-TNGs-treated cells, and was approximately ~5-fold higher than in the control and NH-GQDs group (Fig. 8c, d). The expression of Bcl-2 was slightly repressed by Bax in the FAPEG-TNGs-treated group. However, the Bcl-2 expression in the NH-GQDs-treated group was increased, indicating that apoptosis was repressed. This implies that FAPEG-TNGs accelerated the apoptosis of cells, but NH-GQDs did not. We also examined the expression of Caspase 3 protein, which is responsible for execution of apoptosis. The expression of Caspase 3 in FAPEG-TNGs-treated cells was approximately 11-fold higher than that in the control and NH-GQDs-treated groups. Thus, the FAPEG-TNGs-treated cells showed apoptosis and death. Once again, these results highlighted our finding that FAPEG-TNGs induced the irreversible apoptosis of *HeLa* cells through DNA damage in the nucleus.

### In vivo tumor therapeutics performance of FAPEG-TNGs

**Fig. 9:**
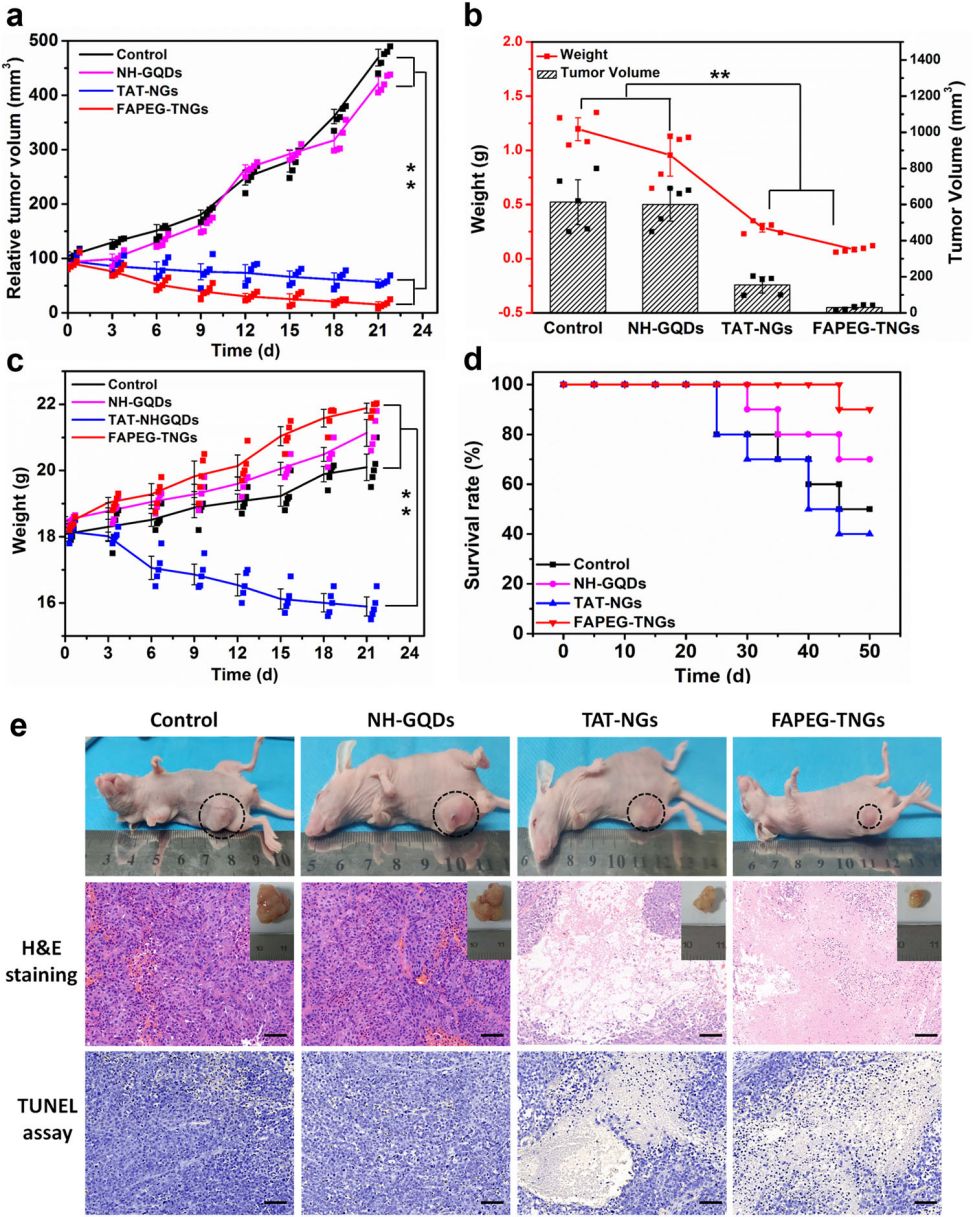
In vivo therapeutic performance in the *HeLa* tumor xenografts. **a** The relative tumor volumes. **b** The volumes and weights of tumor dissected from each group after therapy. **c** The body weights of mice. **d** The survival curves of *HeLa* tumor-bearing mice. **e** Example photos of tumor xenograft mice and histopathology images of dissected *HeLa* tumor xenografts stained for H&E or TUNEL assay. Scale bar: 50 μm.

The therapeutic performance of NH-GQDs derivatives was examined in *HeLa* tumor-bearing nude mice. Saline (control group), NH-GQDs, TAT-NGs (10:1), and FAPEG-TNGs at a dose of 5 mg kg^−1^ were administered intravenously to investigate the therapeutic performance. During 21 days treatment, the relative tumor volume of TAT-NGs (10:1)-treated and FAPEG-TNGs-treated groups were decreased 87.87% and 96.8%, respectively (Fig. 9a). The volume of dissected tumors was found to be decreased by 70% and 91.67% for TAT-NGs (10:1)-treated and FAPEG-TNGs-treated groups, respectively, compared to the control group, and the corresponding variations in tumor weights were 80% and 92%, respectively (Fig. 9b). However, the NH-GQDs alone exhibited negligible anti-tumor effect, indicating the strong therapeutic effect of TAT-NGs and FAPEG-TNGs due to the associated targeting effects. The body weight of the mice was monitored every 3 days. We found that no obvious change in the control, NH-GQDs, and FAPEG-TNGs administration groups (Fig. 9c), indicating negligible systemic toxicity of these nanoparticles. However, the body weight of TAT-NGs-treated mice was significantly decreased. In addition, mice treated with TAT-NGs showed the lowest survival time and survival rate (Fig. 9d) during the treatment. Thus, TAT-NGs showed less tumor-targeting and damaged the normal organs and induced mouse death, though they significantly suppressed tumor grown. The FAPEG-TNGs group showed the longest survival time and highest survival rate, indicating the strong therapeutic effect of FAPEG-TNGs with a high tumor targeting capability and their ability to increase quality of life by selectively killing the tumor cells.

After 21 days, tumors were dissected. The histopathology images of the dissected *HeLa* tumors were stained with hematoxylin and eosin (H&E) (Fig. 9e). The TAT-NGs-treated and FAPEG-TNGs-treated tumor cells all showed obvious destruction, particularly in the nucleus. Cell necrosis, lysis, and pyknosis were observed in these two groups, indicating cell apoptosis by the nanoparticles. The further terminal deoxynucleotidyl transferase-mediated dUTP-biotin nick end labeling (TUNEL) of tumors confirmed cell apoptosis in the TAT-NGs (10:1)-treated and FAPEG-TNGs-treated tumors. In contrast, the control and NH-GQDs groups exhibited remarkable hypercellularity, tight arrangement, and intact tumor shape. Clearly, therefore, these results demonstrated that FAPEG-TNGs provided optimal therapeutic outcomes against solid tumors in vivo.

To further evaluate the pathological damage to major organs by NH-GQDs, TAT-NGs, and FAPEG-TNGs, we dissected the major organs including the heart, liver, spleen, kidney, and lung after treatment and stained them to perform the H&E assay. Little significant damage was observed to the major organs in the NH-GQDs, TAT-NGs, and FAPEG-TNGs treated groups (Supplementary Fig. 11). Finally, we evaluated the blood biochemistry of the treated groups, including indexes of the liver function (alanine aminotransferase (ALT), alkaline phosphatase (ALP), aspartate aminotransferase (AST)) and kidney function (the serum urea nitrogen (BUN), creatinine (Cr)). We found that these indexes were all within the normal reference ranges in the NH-GQDs-treated and FAPEG-TNGs-treated groups (Supplementary Table 1) with no significant difference from the control groups. These results suggest that NH-GQDs and FAPEG-TNGs had little toxicity to the liver, kidney, and the heart during the in vivo treatments. However, the AST, ALT, and BUN levels of the TAT-NGs (10:1)-treated groups were significantly increased compared to those in the control groups (Supplementary Fig. 12). Increase in these indexes can be caused by excessive protein intake, kidney damage, liver damage, or heart failure caused by certain drugs^40^. However, we did not observe pathological alterations of the liver, kidney, or heart in the TAT-NGs (10:1)-treated group (Supplementary Fig. 11), but TAT-NGs (10:1) do induce the serious functional abnormalities, which decreased the survival rate (Fig. 9d).

## Discussion

In this study, we have functionalized GQDs nanoparticles for targeting the tumor cell nucleus to selectively kill the tumor cells through DNA damage. FAPEG-TNGs nanoparticles were developed through the construction of nucleus-targeting TAT peptide-modified GQDs, followed by disulfide bonding with FA-PEG for cancer cell targeting (e.g., *HeLa* cells rather than *L929* cells), and hence selective cancer killing. The incorporation of TAT peptides greatly enhanced the accumulation and uptake of FAPEG-TNGs in the cancer cell nucleus via the binding import receptors importin *α* and *β* on the nuclear membrane. The FA-PEG units increased the dispersion and blood circulation time of their nanoparticles as well as targeting the tumor cells by precisely recognizing the folate receptor on the cancer cell membrane. Indeed, CLSM observation indicated that FAPEG-TNGs were specifically recognized and taken up into the tumor cells. In the cells, TAT-NGs were released from FAPEG-TNGs by the high level of intracellular GSH, and the released TAT-NGs entered into the cell nucleus. As demonstrated by the extracellular study, TAT-NGs adsorbed both ssDNA and supercoiled DNA through the π–π and electrostatic interactions. Thus, TAT-NGs cause the irreversible apoptosis of tumor cells through DNA damage, as demonstrated by the expression of total p53, pi-p53, Bax, and Caspase-3 and repressed the expression of Bcl-2, whereas NH-GQDs were found only in the cytoplasm without the cell death. TAT-NGs without being functionalized with FA-PEG entered into the nuclei of both the cancer and normal cells (e.g., *HeLa* and *L929* cells) to cause the DNA damage in both cases, and hence non-selective cell death. Thus, FAPEG-TNGs exhibited good therapeutic effects for *HeLa* tumor xenografts both in vitro and in vivo. Under the protection of FA-PEG units, FAPEG-TNGs showed high biocompatibility in the blood circulation and precise transport to the tumor area, promoted by the folic acid recognition. The superb therapeutic efficacy arising from the specific interaction between GQD nanoparticles and the cancer cell nucleus, as discovered in this study, should be very useful for the design and development of chemotherapy nanoparticles for clinical applications.

## Methods

### Chemicals and reagents

All chemicals and reagents were purchased from Shanghai Macklin Biochemical Co., Ltd. or Aladdin Co., Ltd in analytic grade. The acetylated TAT (AC-TAT, Ac-NH_2_-CGRKKRRQRRRK-COOH with Mw 1670.03) peptides were purchased from GL Biochem (Shanghai) Ltd. All the cell mediums and additional elements were purchased from Gibco. The ssDNA sequences (Table 1) were chemically synthesized by General Biosystems (Anhui) Co., Ltd. The Supercoiled DNA (*PL*, pX601-cmv-dSaCas9-ADAR2) was purified from *Escherichia coli* DH5α cells. The DNA products were prepared in Tris-HCl buffer (20 mM, pH 7.4) with a proper concentration.

**Table 1:** The sequences of the ssDNA.

	Sequence
*P1*	5′-agtcagtgtggaaaatctctagc-FAM-3′
*HIV1*	5′-gctagagattttccacactgact-3′
*P2*	5′-gcggaggcggtgggggtggacaattgaaaaagcctgaactcaccgcg-3′
*P3*	5′-ttggcctctttgaacagccgcacgccggcatcgatcacgtcccgtgtctcaag-3′

### Fabrication of NH-GQDs

Using citric acid and urea as precursors, NH-GQDs was prepared by hydrothermal method^26,27^. Briefly, citric acid (3 mmol) and urea (9 mmol) were dissolved in 15 mL of DI water to form a clear solution, which was transferred into a Teflon-lined stainless-steel autoclave (20 mL) and heated at 160 °C for 4 h. The crude product was poured into 200 mL of ethanol and collected by centrifugation at 5000 rpm for 5 min. The aqueous suspension of NH-GQDs was dialyzed in DI water (500 Da) for 24 h and filtered with a 0.22 μm filter prior to use. The prepared NH-GQDs were dried at 60 °C for 72 h to determine the final concentration. The calibration curve for NH-GQDs was *y* = 0.158x (mg mL^−1^) for UV-Vis spectroscopy assay at 336 nm.

### Preparation of FAPEG-TNGs

Firstly, the carboxyl groups of AC-TAT peptides were activated by EDC/NHS (N-(3-dimethylaminopropyl)-N′-ethylcarbodiimide hydrochloride (EDC) / hydroxysuccinimide (NHS)) solution^41^ at 37 °C for 1 h. Then NH-GQDs were added into the activated solution containing AC-TAT peptides, and mixed overnight at 37 °C. The mass ratio between AC-TAT peptides and NH-GQDs was set to 1:1, 5:1, and 10:1. The prepared TAT-NGs were purified by a centrifugal filter (3 kDa).

Next, the produced TAT-NGs (10:1) were loaded by FA-PEG-NH_2_ (3400 Da) via the disulfide linkage. First, the amino groups of FA-PEG-NH_2_ were activated by 20 mM of SPDP for 30 min at room temperature. The extra SPDP was removed by a centrifugal filter (3 kDa). Then, TAT-NGs (10:1) were mixed with activated FA-PEG-SPDP solution overnight at 37 °C. During this process, the sulfhydryl group of the TAT peptide connected to the activated FA-PEG-SPDP, developing the disulfide bond. The mass ratio between FA-PEG and TAT-NGs was set as 1:5. The FAPEG-TNGs were further filtered by a centrifugal filter (3 kDa). The FA-PEG standard curve was determined by UV-Vis spectroscopy at 279 nm, and the equation was *y* = 11.512x (mg mL^−1^).

### Characterization

The morphology and structure of the as-prepared NH-GQDs derivatives were observed by HR-TEM (JEOL) and AFM (Bruker Co.). XPS measurements were obtained on a Thermo Escalab 250Xi spectrometer equipped with Al-Kα excitation. The Raman spectrum was recorded on a Thermo Fisher DXR with an excitation wavelength of 532 nm. The FT-IR spectra were conducted on a Thermo Nicolet 6700 spectrometer. UV-Vis absorption spectra were recorded with an Agilent Cary 100 spectrophotometer. Fluorescence spectra were recorded on a Hitachi 7000 fluorescence spectrophotometer at room temperature. The Zeta potentials of NH-GQDs were determined by a Zetasizer Nano ZS 90 analyzer.

### Cell culture

The culture medium of *L929* was DMEM medium, *ARPE-19* cells was in DMEM/F12 medium, and *HeLa* and *OCM-1* cells was RPMI 1640 medium, respectively, which were supplemented with 10% (V/V) qualified fetal bovine serum and gentamicin (50 µg mL^−1^) at 10% CO_2_ and cultured at 37 °C.

### Cell imaging by CLSM

The cells were cultured on 10 × 10 mm slide in a 24-well plate with a density of 30,000 cells per slide. After 24 h, the cell medium was changed to a fresh medium with the addition of NH-GQDs or FAPEG-TNGs (100 μg mL^−1^) and the cells were further cultured for an appropriate time. Gently washed with PBS for 3 times, the cells were fixed by paraformaldehyde (4%). The cell nucleus was dyed by 7-AAD (Invitrogen, Ex/Em: 546/647 nm). Fluorescence imaging of cells was obtained using the CLSM (Zeiss LSM710).

### Cell imaging by TEM

Cells were cultured with NH-GQDs derivatives (100 μg mL^−1^) for 24 h. The cells were fixed with 2.5% glutaraldehyde solution, embedded and sliced into ultra-thin sections, which were observed by TEM^42^ (Hitachi, H-7500).

### Immunofluorescence

The cells were cultured on a 10 × 10 mm slide in a 24-well plate with a density of 30,000 cells per slide for 24 h. The adherent cells were fixed by cold methanol for 5 min at −20 °C and blocked by BSA (3%, W/V) for 1 h at room temperature. Then the antibody of anti-FOLR1 (2 or 5 μg in 1% BSA, SAB1410427, Sigma) was added to each well for 24 h at 37 °C. After hybridization with the FITC-tagged secondary antibody (A16024, Thermo, Ex/Em: 495/515 nm), the cell nuclei were dyed with Hoechst (Beyotime, Ex/Em: 346/460 nm) for 2–5 min at −20 °C. Then, the cell slices were observed by CLSM.

### Cell viability

The cells were cultured in 96-well plates with a density of 3000 cells per well. After 24 h the cell medium was changed to fresh medium with NH-GQDs derivatives for another 24 h. The cell proliferation was evaluated by Cell Counting Kit-8 (CCK-8, Dojindo) and expressed as the percentage with respect to the negative control (which was cultured in the medium without any NH-GQDs derivatives).

The living/dead cells were stained by the Calcein-Am/PI staining (Dojindo). The Calcein-AM/PI staining (Dojindo) was further used for the direct observation of living/dead cells. The cells were cultured in 24-well plates with a density of 30,000 cells per well. After 24 h the cell medium was changed to fresh medium with NH-GQDs derivatives (500 μg mL^−1^) for 24 h. The cells were dyed with the staining solution for 15 min and observed under a fluorescence microscope (OLYMPUS IX81) with green/red fluorescent exciters (Ex/Em of Calcein-AM: 490/515; Ex/Em of PI: 535/617).

### Fluorescence response of NH-GQDs derivatives towards DNA

Both *P1* and *HIV1* were prepared in Tris-HCl buffer (100 mM, pH 7.4). Fluorescence profiles were recorded by a Hitachi F-4600 fluorometer with the Ex and Em slits of 5.0 nm at 700 V.

### Agarose Gel electrophoresis

The ssDNA of *P2* (10 μM), *P3* (10 μM), or supercoiled DNA (*PL*, 2 nM) was prepared in Tris-HCl buffer (pH 7.4) and treated with different concentrations of NH-GQDs or TAT-NGs for 6 h at 25 °C. Each reaction was subjected to electrophoresis on a 2% agarose gel in TBE buffer at 160 V. The agarose gels were stained by SYBR Green I (Thermo) and imaged with the ChemiDoc XRS+ imaging system (Bio-Rad Laboratories, Inc.).

### Intracellular DNA damage assay

The intracellular DNA damaged was determined by the alkaline Comet Assay Reagent Kit (TREVIGEN). The cells were seeded in a 6-well plate with a density of 100,000 cells per well. After 24 h, the cell medium was changed to fresh medium with NH-GQDs derivatives (300 μg mL^−1^) for another 24 h. The negative control was cells cultured in fresh medium. Following the instructions, the DNA was stained with the SYBR Green I and imaged by fluorescence microscope. Using the Comet Assay Software Project (CASP), the percentage of DNA tail was scored. For each sample, approximately 200 random cells were selected.

### Western blot

Cells were cultured in 6-well tissue culture plates with a density of 25,000 cells/well and incubated. After 24 h, the cells were continuously cultured in the well with the addition of NH-GQDs derivatives (300 μg mL^−1^) for 24 h. The harvested cells were lysed in western cracking buffer (Beyotime). The total protein concentration was evaluated by the BCA Protein Assay Kit (Thermo). Western blotting was performed to analyze the expression levels of total p53, phosphorylated p53 (pi-P53), Bax, Bcl-2, and Caspase 3 with their rabbit polyclonal antibodies (Abcam). GAPDH was used as the reference control. Using the Image Lab software (Bio-Rad Laboratories, Inc.) the relative peaks of the bands were calculated, which were normalized to the band of GAPDH.

### Animal experiments

Female BALB/c nude mice aged 4 weeks were purchased from Shanghai SLAC Laboratory Animal Co., Ltd. The animal procedures were all performed at the Laboratory Animal Center of Wenzhou Medical University and following the guidelines of the Laboratory Animal Ethics Committee of Wenzhou Medical University. The in vivo therapeutic evaluation was performed in an animal model of *HeLa* tumor-bearing mice. The mice were randomly assigned into four groups (*n* = 10): control (100 μL, saline), NH-GQDs (100 μL, 5 mg kg^−1^), TAT-NGs (10:1) (100 μL, 5 mg kg^−1^), and FAPEG-TNGs (100 μL, 5 mg kg^−1^). When the tumors grew to 20 mm^3^, the corresponding drugs were injected intravenously into the nude mice in three rounds during the first 7 days. For every other day, the body weights and tumor volumes of the mice were measured. After 21 days treatment, half of the nude mice were sacrificed for anatomical and histopathological analysis. The residue nude mice were continuously fed until 50 days to evaluate the survival rates.

After the treatment, a blood sample was collected from each mouse in a sodium EDTA anticoagulant tube for blood biochemistry testing. The serum levels of ALT, AST, and ALP were determined to evaluate the liver function. Kidney function was determined by measuring BUN and Cr levels.

### Statistic and reproducibility

All data were presented as the means of six parallel tests and standard derivations. The skewness and kurtosis values of all data were calculated by SPSS. For normally distributed data, the significance (*p* value) was evaluated by unpaired, two-tailed Student’s *t*-tests with unequal variance, which was denoted by the symbol “*” compared with the negative control (* ≤ 0.05, ** ≤ 0.01, ^***^ ≤ 0.001). The *p* value ≤ 0.05 was considered as significant.

### Reporting summary

Further information on research design is available in the Nature Research Reporting Summary linked to this article.

## Supplementary information

Supplementary information.

Description of Additional Supplementary Files.

Supplementary Video 1.

Supplementary Video 2.

Supplementary Video 3.

Supplementary data.

Reporting summary.

## Data Availability

The uncropped blots are shown in Supplementary Information. The Source data shown in figures are provided in the file of Supplementary Data. All other data in the published article or supplementary files are available within the manuscript files.
