# Supplementary material for: Biocompatible nucleus-targeted graphene quantum dots for selective killing of cancer cells via DNA damage
Source: Commun Biol. 2021 Feb 16;4:214. doi: 10.1038/s42003-021-01713-1 (PMC7886873; doi:10.1038/s42003-021-01713-1)
Supplement: Supplementary file 1 — Supplementary information. [file 42003_2021_1713_MOESM1_ESM.pdf]

Supplementary Information

Biocompatible Nucleus-Targeted Graphene Quantum Dots for Selective Killing of Cancer Cells Via DNA Damage

Lei Qi[a], Tonghe Pan[a], Liling Ou[a], Zhiqiang Ye[a], Chunlei Yu[a], Bijun Bao[a], Zixia Wu[a], Dayong Cao[b,*], Liming Dai[a,c,*]

[a]State key Laboratory of Ophthalmology, Optometry and Visual Science, Institute of Advanced Materials for Nano-Bio Applications, School of Ophthalmology and Optometry, School of Biomedical Engineering, Wenzhou Medical University, 270 Xueyuanxi Road, Wenzhou 325027, China

[b]Department of General Surgery, The First Hospital of Qiqihar, Affiliated Qiqihar Hospital, Southern Medical University, Qiqihar 161005, China

[c]Center of Advanced Science and Engineering for Carbon (Case4Carbon), Case Western Reserve University, Cleveland, OH 44106, United States

[†]Authors with equal contribution

***Corresponding Author:** L.D. (email: liming.dai@hotmail.com)

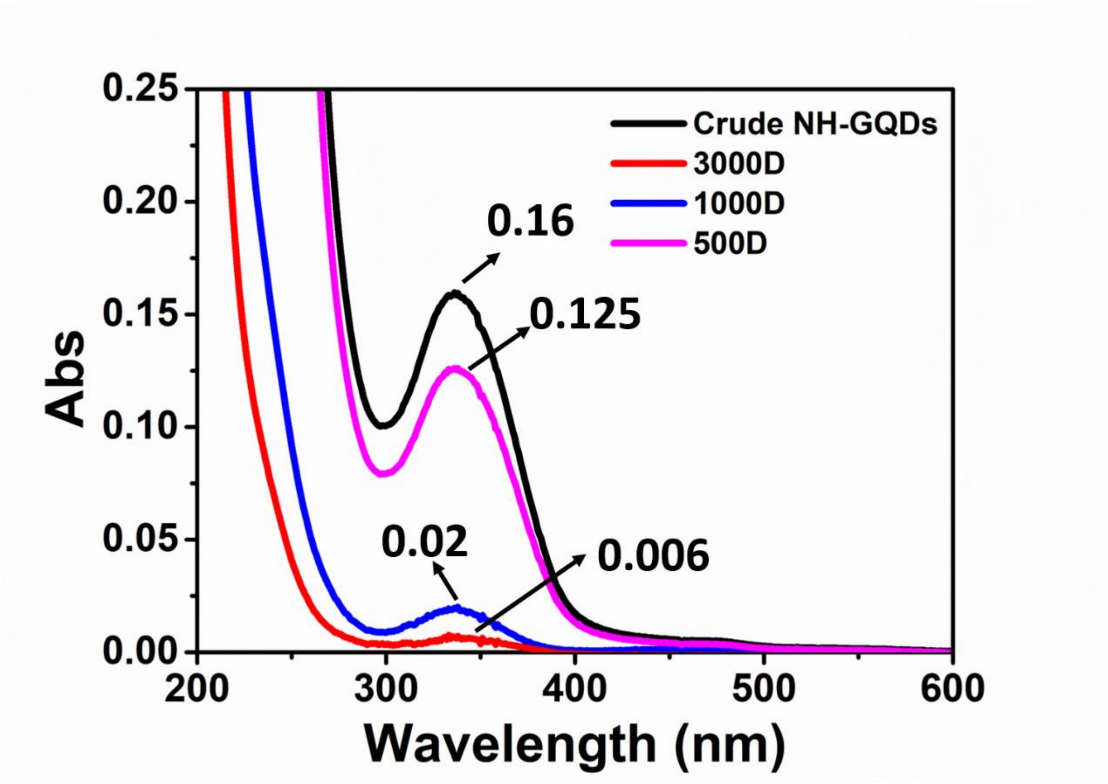

Supplementary Fig. 1. The UV-Vis spectra of crude NH-GQDs and purified NH-GQDs.

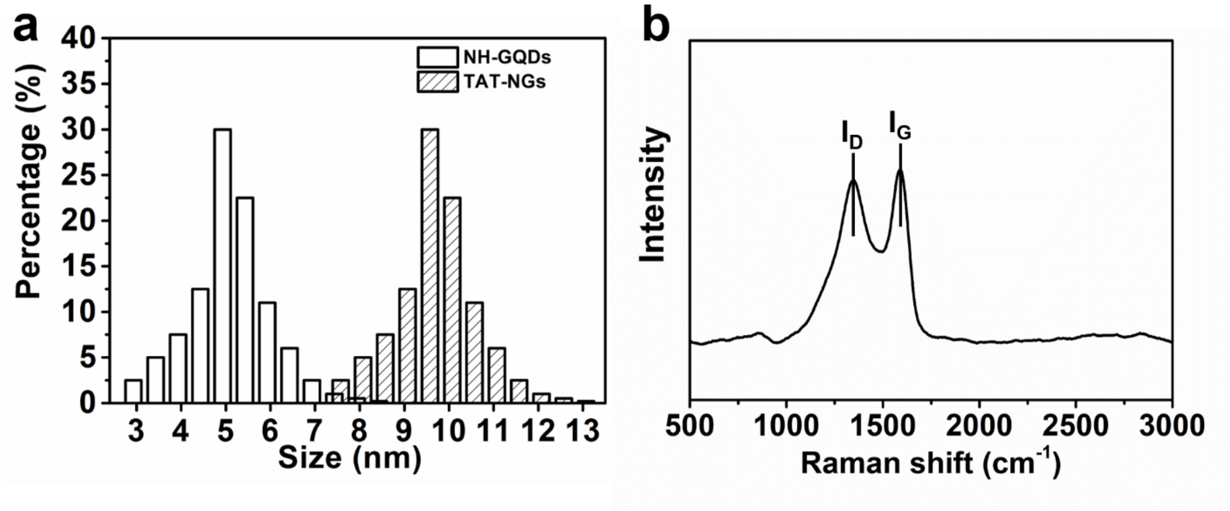

Supplementary Fig. 2. (**a**) The size distribution of NH-GQDs and FAPEG-TNGs. (**b**) The Raman spectrum of NH-GQDs.

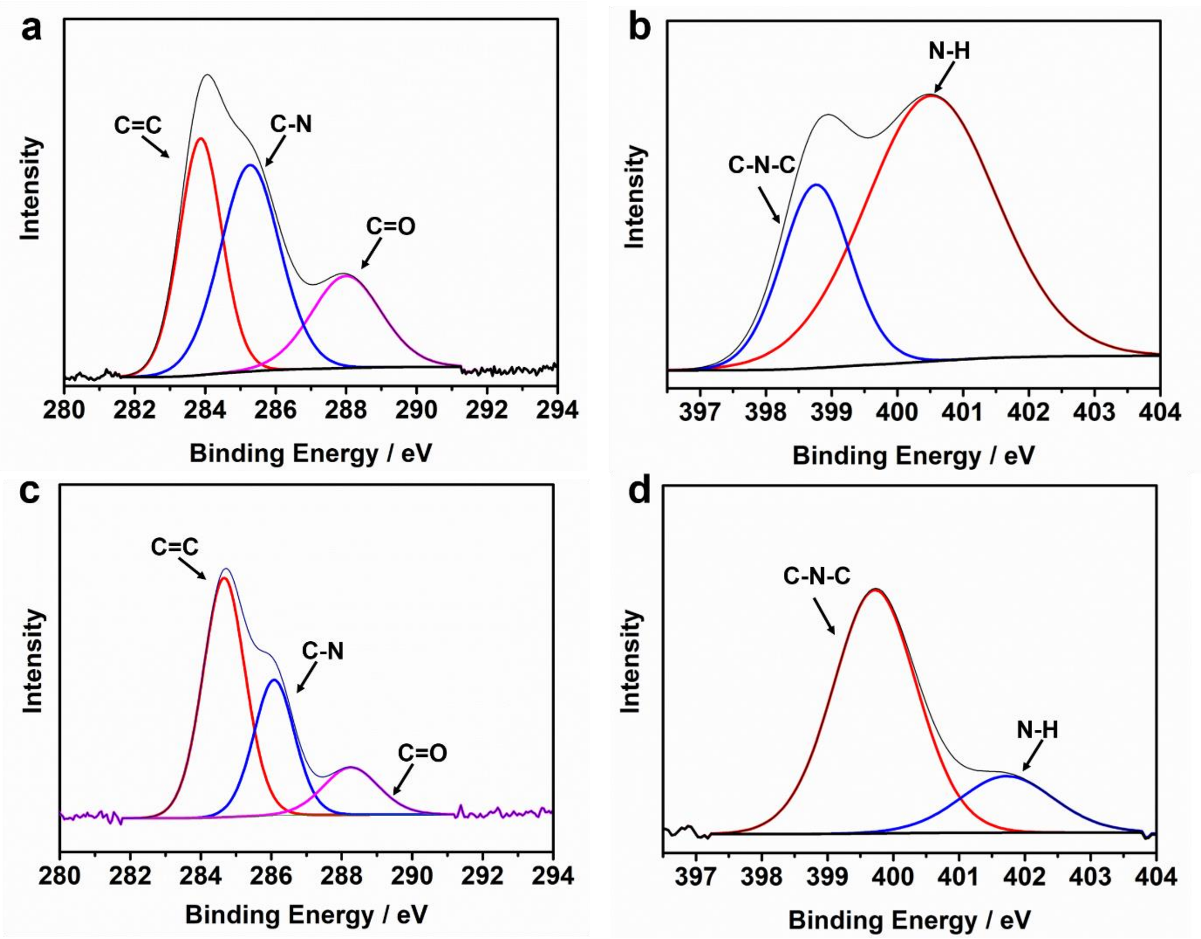

Supplementary Fig. 3. XPS spectra of NH-GQDs and TAT-NGs (10:1). (**a**) XPS C1 spectra of NH-GQDs. (**b**) XPS N1 spectra of NH-GQDs. (**c**) XPS C1 spectra of TAT-NGs. (**d**) XPS N1 spectra of TAT-NGs (10:1).

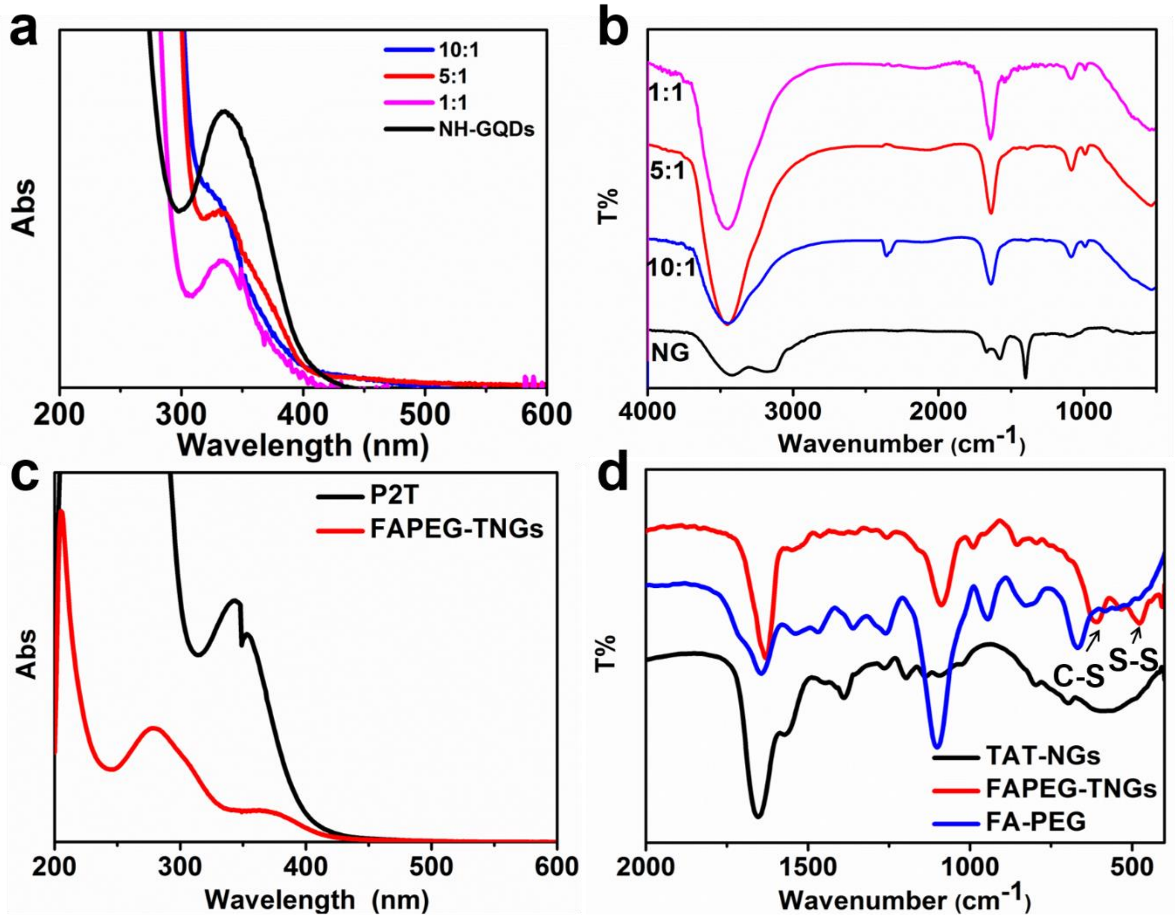

Supplementary Fig. 4. (**a**) The UV spectra of TAT-NGs with different mass ratio of AC-TAT to NH-GQDs (1:1, 5:1 and 10:1, see text) and NH-GQDs. (**b**) The FT-IR spectra of TAT-NGs with different mass ratio of AC-TAT to NH-GQDs (1:1, 5:1 and 10:1, see text) and NH-GQDs (NG). (**c**) The UV-Vis spectra of FAPEG-TNGs and the reaction by-product of P2T. (**d**) The FT-IR spectra of TAT-NGs, FAPEG-TNGs and FA-PEG.

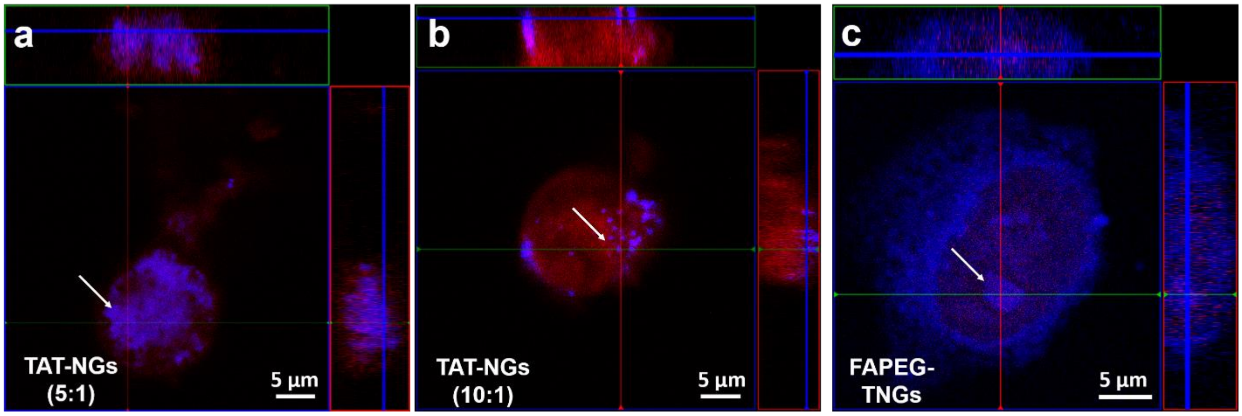

Supplementary Fig. 5. The 3D fluorescence images of HeLa cells incubated with (**a**) TAT-NGs (5:1), (**b**) TAT-NGs (10:1), and (**c**) FAPEG-TNGs.

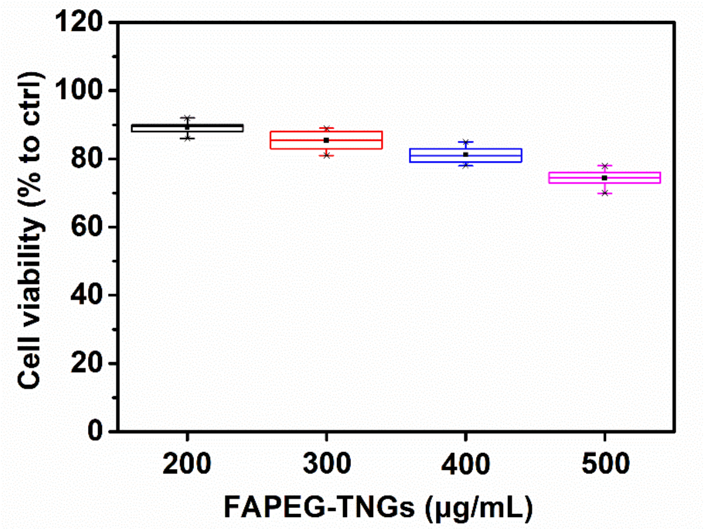

Supplementary Fig. 6. The cell viability of L929 after treated with different concentrations of FAPEG-TNGs for 72 h.

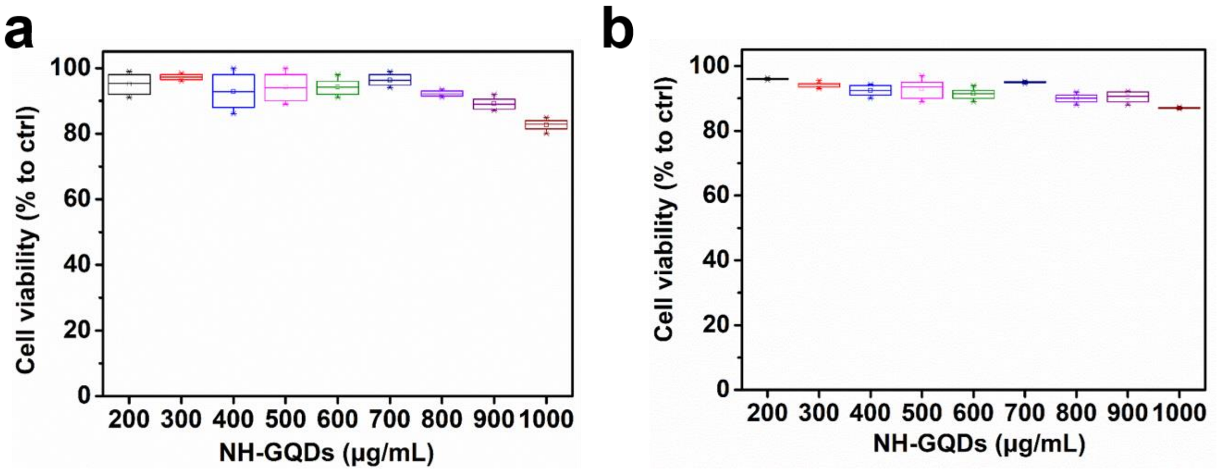

Supplementary Fig. 7. The cell viability of (**a**) HeLa and (**b**) L929 cells after treated with different concentrations of NH-GQDs for 24 h.

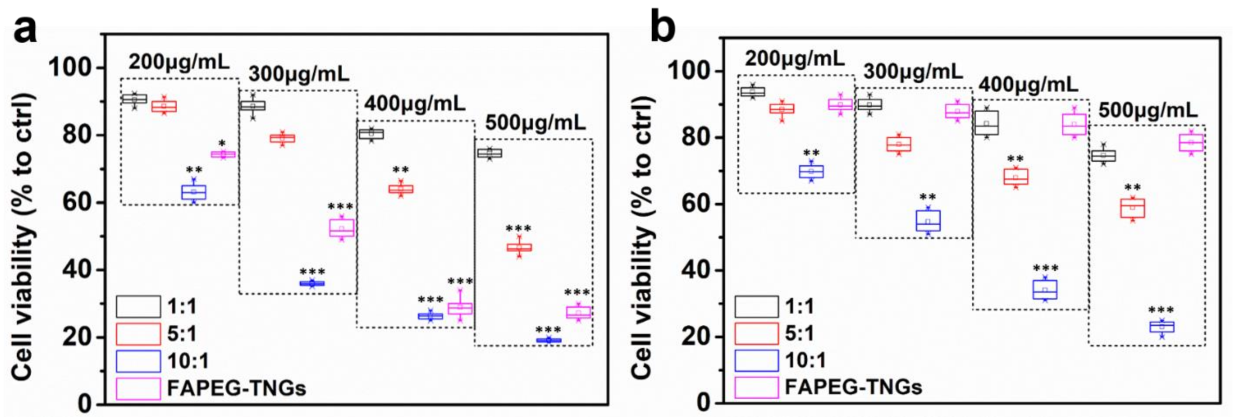

Supplementary Fig. 8. CCK-8 assay determined the cell viability of (**a**) OCM-1 and (**b**) ARPE-19 cells after treated with TAT-NGs and FAPEG-TNGs for 24 h.

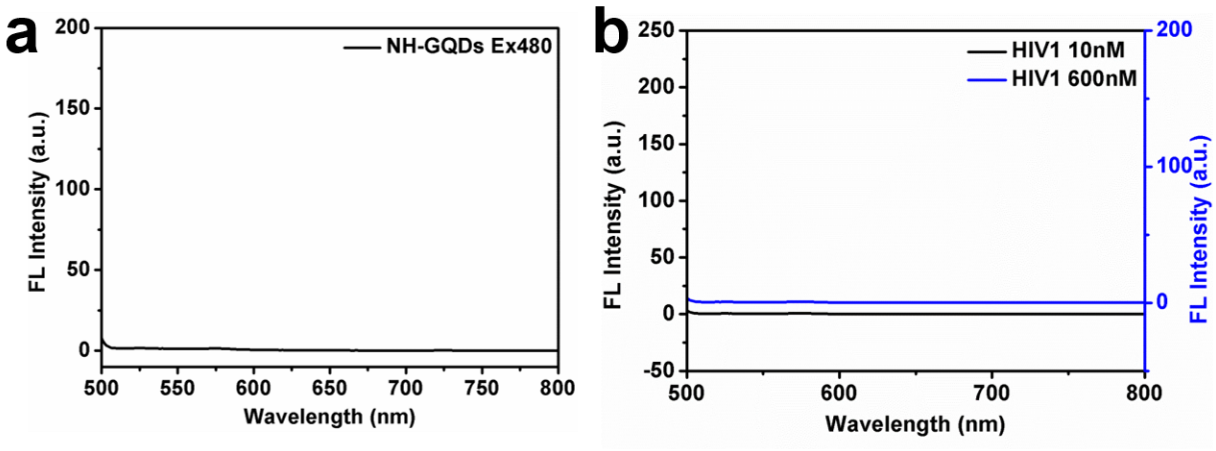

Supplementary Fig. 9. (**a**) The fluorescence emission spectra of NH-GQDs excited at 480 nm. (**b**) The fluorescence emission spectra of HIV1 excited at 480 nm.

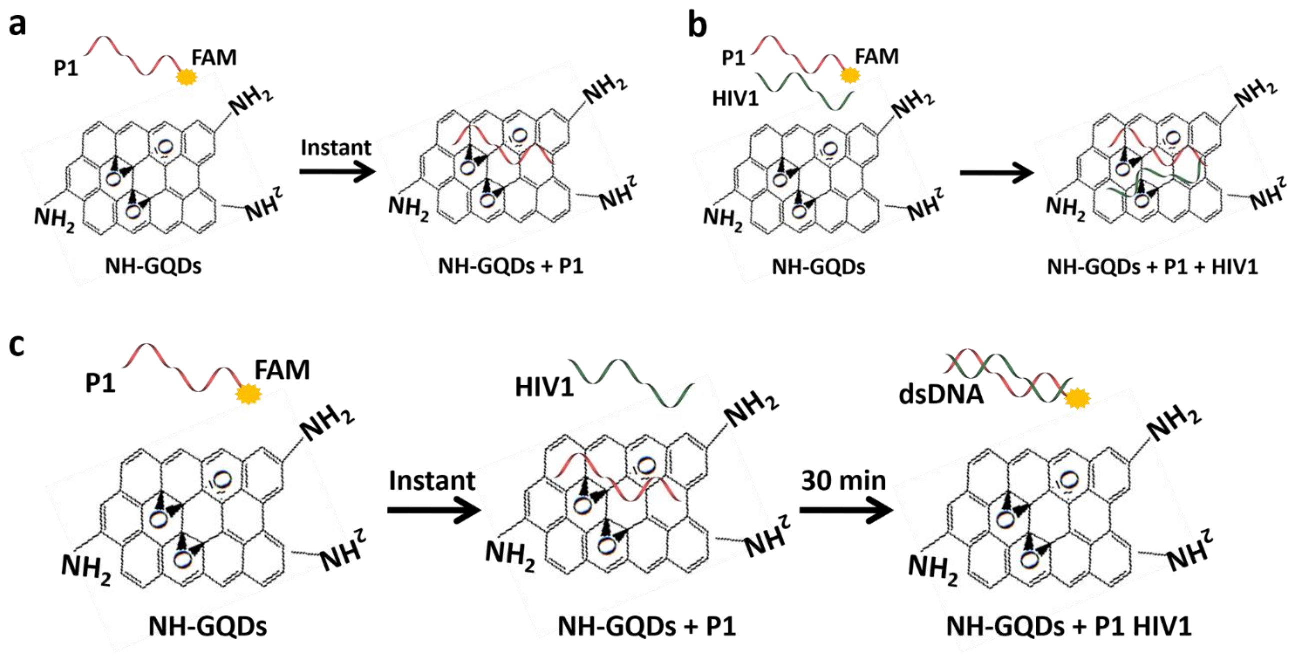

Supplementary Fig. 10. Schematic representation of the target-induced fluorescence change of the P1-FAM mixed (**a**) NH-GQDs, (**b**) the mixture of P1-FAM, complementary P1 (HIV1) and NH-GQDs, (**c**) the formation & release of the dsDNA from NH-GQDs.

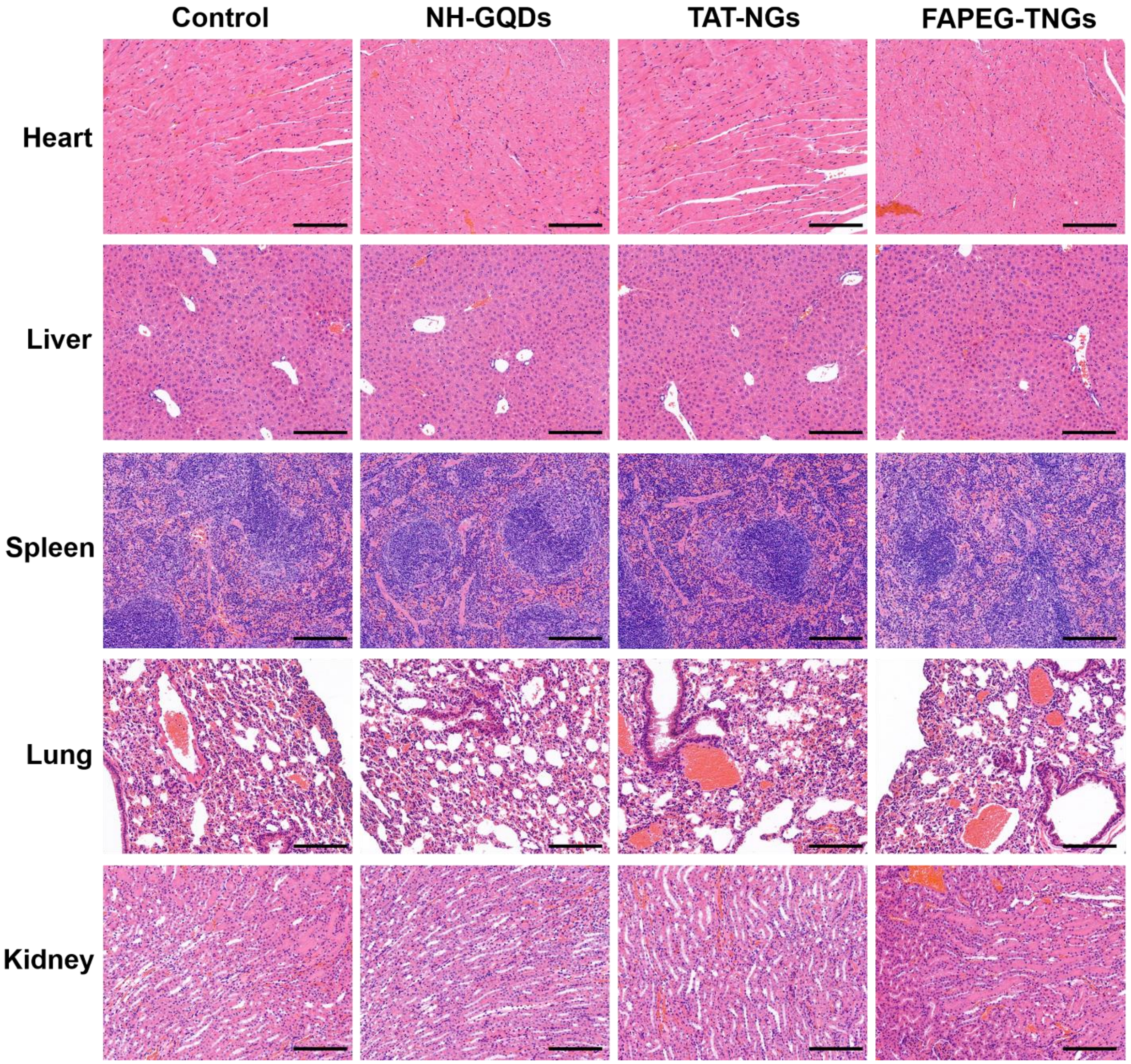

Supplementary Fig. 11. H&E staining of the heart, liver, spleen, lung and kidney after the treatment (Scale bar, 50 μm).

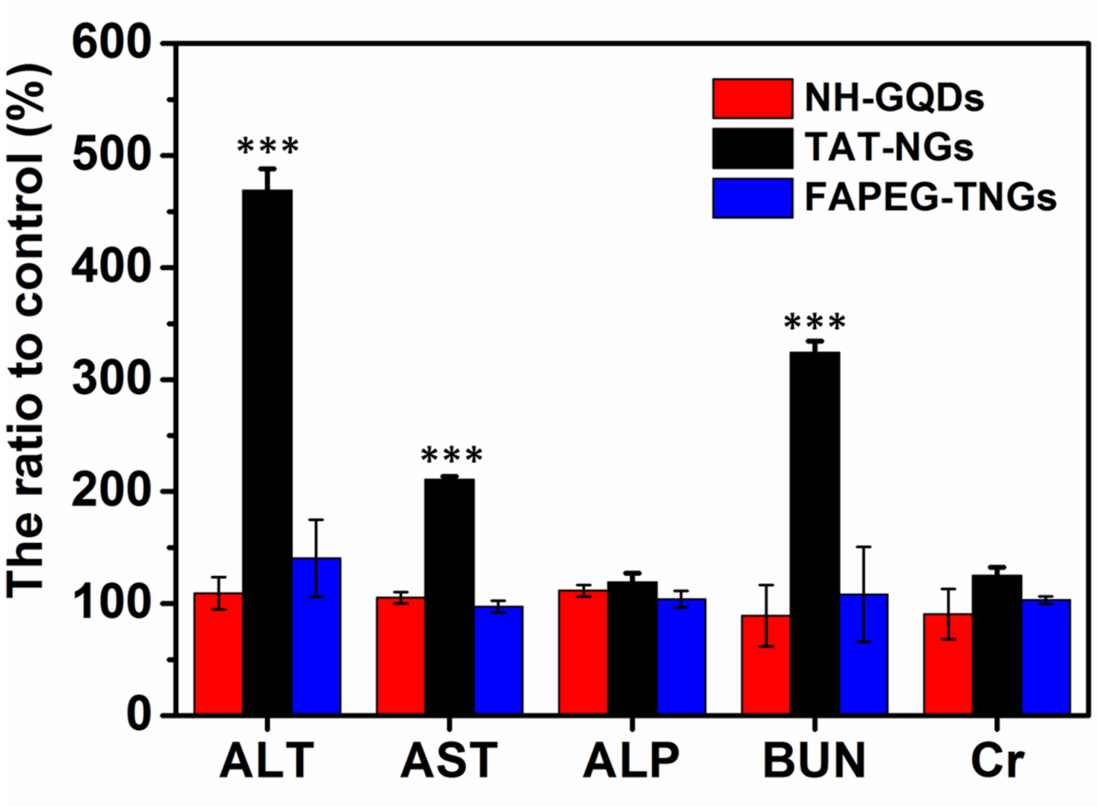

Supplementary Fig. 12. The relative ratio of blood indexes of NH-GQDs, TAT-NGs, and FAPEG-TNGs treated groups comparing to the control group.

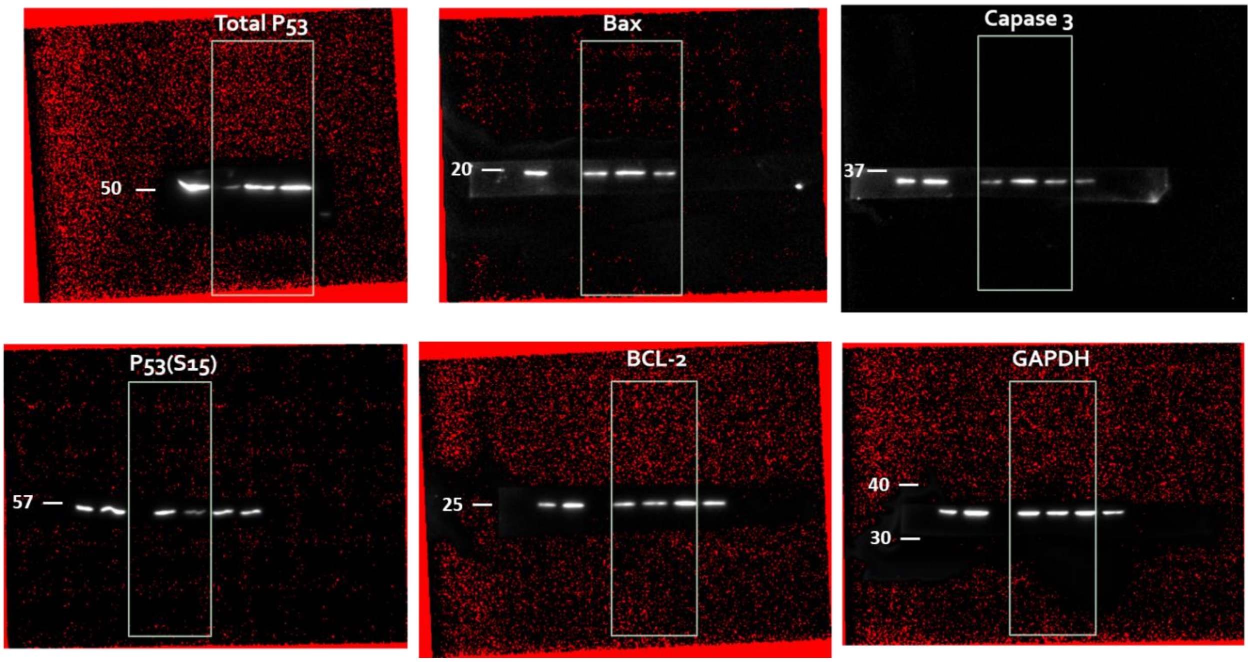

Supplementary Fig. 13. Uncropped WB images for the data presented in the main figures of Fig. 8.

Supplementary Table 1. Biochemical blood analysis of the NH-GQDs, TAT-NGs, and FAPEG-TNGs -treated mice. Data are Presented as Mean ± SD.

	Control	NH-GQDs	TAT-NGs	FAPEG-TNGs	Reference
ALT (U·L-1)	32 ± 2.5	35 ± 3.6	150 ± 4.8	45 ± 8.6	27~78
AST (U·L-1)	190 ± 10.4	200 ± 5.4	400 ± 3.2	185 ± 5.4	50~215
ALP (U·L-1)	130 ± 8.4	145 ± 4.2	155 ± 6.7	135 ± 6.2	63~178
BUN (mg·dL-1)	37 ± 1.2	33 ± 3.3	120 ± 12	40 ± 5.1	18~45
Cr (μmol·L-1)	32 ± 3.6	29 ± 8.1	40 ± 2.7	33 ± 1.2	27~44
