# Supplementary material for: Biocompatible nucleus-targeted graphene quantum dots for selective killing of cancer cells via DNA damage
Source: Commun Biol. 2021 Feb 16;4:214. doi: 10.1038/s42003-021-01713-1 (PMC7886873; doi:10.1038/s42003-021-01713-1)
Supplement: Supplementary file 2 — Description of Additional Supplementary Files. [file 42003_2021_1713_MOESM2_ESM.pdf]

Description of Additional Supplementary Files

File Name: Supplementary Video 1
Description: The 3D fluorescence images of HeLa cells incubated with TAT-NGs (5:1)

File Name: Supplementary Video 2
Description: The 3D fluorescence images of HeLa cells incubated with TAT-NGs (10:1)

File Name: Supplementary Video 3
Description: The 3D fluorescence images of HeLa cells incubated with TAT-NGs (10:1)

File Name: Supplementary Data 1
Description: The source Data of Fig. 3f; Fig. 6a; Fig. 8b; Fig. 8d; Fig. 9a; Fig. 9b; Fig.9c; Fig. 9d.
