# Supplementary material for: Biocompatible nucleus-targeted graphene quantum dots for selective killing of cancer cells via DNA damage
Source: Commun Biol. 2021 Feb 16;4:214. doi: 10.1038/s42003-021-01713-1 (PMC7886873; doi:10.1038/s42003-021-01713-1)
Supplement: Supplementary file 6 — Supplementary data. [file 42003_2021_1713_MOESM6_ESM.pdf]

**Fig. 3f**

| | NG | 1:1 | 5:1 | 10:1 | FAPEG-TNGs |
|---|---|---|---|---|---|
| Relative fluorescence (%) | 1.2 | 15 | 60 | 90 | 85 |
| | 1 | 20 | 62 | 98 | 91 |
| | 1.3 | 18 | 61 | 82 | 90 |
| | 0.9 | 16 | 61.5 | 96 | 89 |
| | 0.95 | 17 | 60.5 | 95 | 88 |
| | 1.1 | 10 | 58 | 85 | 79 |
| | 1.4 | 12 | 59.8 | 84 | 80 |
| | 1.5 | 13 | 58.5 | 88 | 82 |

**Fig. 6a-HeLa**

| | 200 µg/mL | | | | 300 µg/mL | | | | 400 µg/mL | | | | 500 µg/mL | | | | |
|---|---|---|---|---|---|---|---|---|---|---|---|---|---|---|---|---|---|
| | 1:1 | 5:1 | 10:1 | FAPEG-TNGs | 1:1 | 5:1 | 10:1 | FAPEG-TNGs | 1:1 | 5:1 | 10:1 | FAPEG-TNGs | 1:1 | 5:1 | | 10:1 | FAPEG-TNGs |
| Cell viability (% to control) | 90.8 | 89.4 | 70 | 74.75 | 87.9 | 79.7 | 50 | 55 | 80.69 | 64 | 26.7 | 22 | 74.82 | 47.68 | | 19.1 | 20 |
| | 91 | 90 | 66 | 75 | 83 | 80 | 49.2 | 55.3 | 81.6 | 66 | 27 | 22.4 | 75 | 49 | | 20 | 22 |
| | 92 | 88 | 68 | 74.5 | 85 | 81 | 49.8 | 55.1 | 81 | 66.5 | 27.9 | 22.2 | 75.1 | 50 | | 18 | 21.5 |
| | 91.5 | 91 | 72 | 75.1 | 90 | 78.4 | 50.8 | 54.8 | 80.7 | 62 | 25.7 | 21.6 | 74.5 | 46 | | 20.1 | 21.8 |
| | 89 | 90.5 | 73.5 | 74.2 | 91 | 79 | 50.2 | 54.7 | 79.7 | 61.5 | 26 | 21.8 | 74.3 | 45.5 | | 19.8 | 19.5 |
| | 88.6 | 88.5 | 74 | 74.4 | 90.5 | 80.3 | 50.4 | 55 | 80 | 62.5 | 27 | 22.1 | 75.2 | 46.5 | | 18.5 | 18 |

**Fig. 6a-L929**

| | 200 µg/mL | | | | 300 µg/mL | | | | 400 µg/mL | | | | 500 µg/mL | | | |
|---|---|---|---|---|---|---|---|---|---|---|---|---|---|---|---|---|
| | 1:1 | 5:1 | 10:1 | FAPEG-TNGs | 1:1 | 5:1 | 10:1 | FAPEG-TNGs | 1:1 | 5:1 | 10:1 | FAPEG-TNGs | 1:1 | 5:1 | 10:1 | FAPEG-TNGs |
| Cell viability (% to control) | 96 | 86 | 73 | 92 | 92 | 75 | 60 | 90 | 90 | 70 | 40 | 88 | 80 | 62 | 30 | 85 |
| | 95 | 85 | 72 | 92.2 | 92.5 | 75.3 | 60.8 | 90.1 | 90.1 | 70.25 | 41 | 88.14 | 80.2 | 62.2 | 31 | 85.28 |
| | 95.5 | 85.5 | 72.5 | 92.1 | 92.3 | 75.2 | 60.4 | 90.06 | 90.3 | 70.1 | 41.2 | 88.1 | 80.15 | 62.1 | 31.2 | 85.2 |
| | 96.5 | 87 | 74 | 91.8 | 91.5 | 75.1 | 60.6 | 89.9 | 89.9 | 70.2 | 38.8 | 88.08 | 80.1 | 62.27 | 29.8 | 84.8 |
| | 97 | 86.5 | 73.5 | 91.9 | 91.8 | 74.8 | 59.2 | 89.8 | 89.7 | 69.75 | 39 | 87.9 | 79.8 | 61.85 | 28.8 | 84.78 |
| | 96.8 | 86.8 | 73.8 | 92.24 | 91.7 | 74.7 | 59.8 | 90 | 89.8 | 69.85 | 39.7 | 87.8 | 79.9 | 61.8 | 30.5 | 85.1 |

**Fig. 8b**

| | Control | NH-GQDs | TAT-NGs (10:1) | FAPEG-TNGs |
|---|---|---|---|---|
| Tail DNA (100% to control) | 0.2 | 0.45 | 7.6 | 8.7 |
| | 0.25 | 0.47 | 7.7 | 9 |
| | 0.23 | 0.46 | 7.65 | 8.8 |
| | 0.21 | 0.455 | 7.62 | 8.9 |
| | 0.15 | 0.43 | 7.5 | 8.4 |
| | 0.16 | 0.44 | 7.55 | 8.5 |
| | 0.18 | 0.435 | 7.58 | 8.6 |

**Fig. 8d**

| | NC | FAPEG-TNGs | NH-GQDs |
|---|---|---|---|
| Total p53 | 1 | 3.5 | 3 |
| pi-p53 | 1 | 12 | 1 |
| Bax | 1 | 5 | 1.2 |
| Bcl-2 | 1 | 0.8 | 2 |
| Capase 3 | 1 | 10 | 1.2 |

**Fig.9a**

| | Tumor volume ($mm^3$) | | | | | | | Tumor volume ($mm^3$) | | | | | | |
|---|---|---|---|---|---|---|---|---|---|---|---|---|---|---|
| Days | control | | | | | Mean | Er | NH-GQDs | | | | | Mean | Er |
| 0 | 91 | 96 | 105 | 110 | 118 | 104 | 8.4 | 82 | 88 | 90 | 95 | 110 | 93 | 7.6 |
| 3 | 121 | 125 | 130 | 135 | 137 | 129.6 | 5.28 | 85 | 90 | 100 | 105 | 115 | 99 | 9.2 |
| 6 | 134 | 140 | 156 | 160 | 166 | 151.2 | 11.36 | 121 | 123 | 125 | 135 | 145 | 129.8 | 8.16 |
| 9 | 167 | 172 | 180 | 190 | 193 | 180.4 | 8.88 | 148 | 150 | 165 | 170 | 175 | 161.6 | 10.08 |
| 12 | 220 | 244 | 250 | 260 | 270 | 248.8 | 13.44 | 250 | 262 | 265 | 270 | 277 | 264.8 | 7.04 |
| 15 | 248 | 262 | 277 | 298 | 310 | 279 | 20 | 281 | 285 | 290 | 295 | 310 | 292.2 | 8.24 |
| 18 | 335 | 356 | 360 | 375 | 380 | 361.2 | 13.04 | 298 | 300 | 302 | 331 | 355 | 317.2 | 20.64 |
| 21 | 440 | 460 | 476 | 480 | 490 | 469.2 | 15.36 | 405 | 410 | 420 | 436 | 438 | 421.8 | 12.16 |

| | Tumor volume ($mm^3$) | | | | | | | Tumor volume ($mm^3$) | | | | | | |
|---|---|---|---|---|---|---|---|---|---|---|---|---|---|---|
| Days | TAT-NGs | | | | | Mean | Er | FAPEG-TNGs | | | | | Mean | Er |
| 0 | 82 | 85 | 92 | 105 | 115 | 95.8 | 11.36 | 80 | 85 | 88 | 95 | 112 | 92 | 9.2 |
| 3 | 70 | 75 | 88 | 92 | 105 | 86 | 10.8 | 68 | 70 | 75 | 80 | 88 | 76.2 | 6.24 |
| 6 | 64 | 68 | 78 | 92 | 102 | 80.8 | 12.96 | 42 | 45 | 51 | 60 | 65 | 52.6 | 7.92 |
| 9 | 45 | 70 | 75 | 80 | 108 | 75.6 | 14.72 | 25 | 36 | 38 | 45 | 55 | 39.8 | 8.16 |
| 12 | 50 | 60 | 80 | 88 | 90 | 73.6 | 14.88 | 23 | 25 | 30 | 35 | 39 | 30.4 | 5.28 |
| 15 | 51 | 55 | 70 | 75 | 81 | 66.4 | 10.72 | 12 | 15 | 28 | 35 | 38 | 25.6 | 9.68 |
| 18 | 43 | 50 | 67 | 70 | 78 | 61.6 | 12.08 | 14 | 18 | 22 | 25 | 25 | 20.8 | 3.84 |
| 21 | 50 | 52 | 55 | 58 | 69 | 56.8 | 5.36 | 8 | 12 | 15 | 18 | 25 | 15.6 | 4.72 |

**Fig. 9b weight**

| | Weight ( g) | | | | | Mean | Er |
|---|---|---|---|---|---|---|---|
| Control | 1.3 | 1.05 | 1.2 | 1.08 | 1.35 | 1.196 | 0.1048 |
| NH-GQDs | 0.65 | 0.78 | 1.13 | 1.1 | 1.12 | 0.956 | 0.1928 |
| TAT-NGs | 0.23 | 0.35 | 0.3 | 0.31 | 0.24 | 0.286 | 0.0408 |
| FAPEG-TNGs | 0.06 | 0.072 | 0.08 | 0.095 | 0.12 | 0.0854 | 0.01768 |

**Fig. 9b Tumor Volume**

| | Tumor Volume ($mm^3$) | | | | | Mean | Er |
|---|---|---|---|---|---|---|---|
| Control | 730 | 450 | 620 | 465 | 800 | 613 | 124.4 |
| NH-GQDs | 450 | 520 | 690 | 660 | 680 | 600 | 92 |
| TAT-NGs | 97 | 203 | 185 | 190 | 100 | 155 | 45.2 |
| FAPEG-TNGs | 12 | 20 | 35 | 44 | 42 | 30.6 | 11.68 |

**Fig.9c**

| | Weight ( g) | | | | | | | Weight ( g) | | | | | | |
|---|---|---|---|---|---|---|---|---|---|---|---|---|---|---|
| Days | control | | | | | Mean | Er | NH-GQDs | | | | | Mean | Er |
| 0 | 17.8 | 17.9 | 18 | 18.2 | 18.5 | 18.08 | 0.216 | 18.3 | 18.45 | 18.52 | 18.6 | 18.65 | 18.504 | 0.1032 |
| 3 | 17.5 | 18 | 18.5 | 18.7 | 18.8 | 18.3 | 0.44 | 18.4 | 18.53 | 18.85 | 18.9 | 19.2 | 18.776 | 0.2488 |
| 6 | 18.2 | 18.4 | 18.45 | 18.5 | 19 | 18.51 | 0.196 | 18.7 | 18.9 | 19.1 | 19.25 | 19.3 | 19.05 | 0.2 |
| 9 | 18.2 | 18.8 | 18.9 | 19 | 19.5 | 18.88 | 0.304 | 18.8 | 19 | 19.3 | 19.5 | 19.8 | 19.28 | 0.304 |
| 12 | 18.7 | 18.9 | 19 | 19.2 | 19.5 | 19.06 | 0.232 | 19.2 | 19.5 | 19.7 | 19.8 | 19.8 | 19.6 | 0.2 |
| 15 | 18.8 | 19 | 19.15 | 19.2 | 20 | 19.23 | 0.308 | 19.8 | 19.9 | 20 | 20.1 | 20.5 | 20.06 | 0.192 |
| 18 | 19.4 | 19.8 | 20 | 20.1 | 20.15 | 19.89 | 0.232 | 20.1 | 20.35 | 20.5 | 20.5 | 21 | 20.49 | 0.212 |
| 21 | 19.5 | 19.8 | 20 | 20.2 | 21 | 20.1 | 0.4 | 20.6 | 20.8 | 21 | 21.5 | 21.8 | 21.14 | 0.408 |

| | Weight ( g) | | | | | | | Weight ( g) | | | | | | |
|---|---|---|---|---|---|---|---|---|---|---|---|---|---|---|
| Days | TAT-NGs | | | | | Mean | Er | FAPEG-TNGs | | | | | Mean | Er |
| 0 | 17.8 | 18 | 18.22 | 18.3 | 18.5 | 18.164 | 0.2112 | 18.2 | 18.31 | 18.35 | 18.4 | 18.6 | 18.372 | 0.1024 |
| 3 | 17.8 | 17.92 | 18 | 18.05 | 18.3 | 18.014 | 0.1288 | 18.8 | 18.91 | 19 | 19.15 | 19.3 | 19.032 | 0.1544 |
| 6 | 16.5 | 16.8 | 17 | 17.2 | 17.8 | 17.06 | 0.352 | 18.75 | 19 | 19.35 | 19.5 | 19.8 | 19.28 | 0.324 |
| 9 | 16.48 | 16.52 | 16.8 | 17 | 17.5 | 16.86 | 0.312 | 19 | 19.5 | 19.83 | 20.3 | 20.5 | 19.826 | 0.4608 |
| 12 | 16 | 16.3 | 16.5 | 16.9 | 17 | 16.54 | 0.328 | 19.7 | 19.9 | 20 | 20.2 | 20.9 | 20.14 | 0.328 |
| 15 | 15.7 | 15.9 | 16 | 16.2 | 16.8 | 16.12 | 0.304 | 20.5 | 20.9 | 21 | 21.3 | 21.5 | 21.04 | 0.288 |
| 18 | 15.6 | 15.72 | 16.2 | 16 | 16.5 | 16.004 | 0.2768 | 21 | 21.5 | 21.8 | 21.82 | 21.8 | 21.584 | 0.2672 |
| 21 | 15.5 | 15.66 | 15.8 | 16 | 16.5 | 15.892 | 0.2864 | 21.6 | 21.8 | 22 | 22 | 22.03 | 21.886 | 0.1488 |

**Fig.9d**

| | Survival rate (%) | | | |
|---|---|---|---|---|
| Days | Control | NH-GQDs | TAT-NGs | FAPEG-TNGs |
| 0 | 100 | 100 | 100 | 100 |
| 5 | 100 | 100 | 100 | 100 |
| 10 | 100 | 100 | 100 | 100 |
| 15 | 100 | 100 | 100 | 100 |
| 20 | 100 | 100 | 100 | 100 |
| 25 | 80 | 100 | 80 | 100 |
| 30 | 80 | 90 | 70 | 100 |
| 35 | 70 | 80 | 70 | 100 |
| 40 | 60 | 80 | 50 | 100 |
| 45 | 50 | 70 | 40 | 90 |
| 50 | 50 | 70 | 40 | 90 |
