# Supplementary material for: Biocompatible nucleus-targeted graphene quantum dots for selective killing of cancer cells via DNA damage
Source: Commun Biol. 2021 Feb 16;4:214. doi: 10.1038/s42003-021-01713-1 (PMC7886873; doi:10.1038/s42003-021-01713-1)
Supplement: Supplementary file 7 — Reporting summary. [file 42003_2021_1713_MOESM7_ESM.pdf]

nature research

Corresponding author(s): DBPR COMMSBIO-20-1408B

Last updated by author(s): Oct 12, 2020

Reporting Summary

Nature Research wishes to improve the reproducibility of the work that we publish. This form provides structure for consistency and transparency in reporting. For further information on Nature Research policies, see our Editorial Policies and the Editorial Policy Checklist.

Statistics

For all statistical analyses, confirm that the following items are present in the figure legend, table legend, main text, or Methods section.

n/a	Confirmed	
☐	☒	The exact sample size (*n*) for each experimental group/condition, given as a discrete number and unit of measurement
☐	☒	A statement on whether measurements were taken from distinct samples or whether the same sample was measured repeatedly
☐	☒	The statistical test(s) used AND whether they are one- or two-sided<br>*Only common tests should be described solely by name; describe more complex techniques in the Methods section.*
☐	☒	A description of all covariates tested
☐	☒	A description of any assumptions or corrections, such as tests of normality and adjustment for multiple comparisons
☐	☒	A full description of the statistical parameters including central tendency (e.g. means) or other basic estimates (e.g. regression coefficient) AND variation (e.g. standard deviation) or associated estimates of uncertainty (e.g. confidence intervals)
☐	☒	For null hypothesis testing, the test statistic (e.g. *F*, *t*, *r*) with confidence intervals, effect sizes, degrees of freedom and *P* value noted<br>*Give P values as exact values whenever suitable.*
☐	☒	For Bayesian analysis, information on the choice of priors and Markov chain Monte Carlo settings
☐	☒	For hierarchical and complex designs, identification of the appropriate level for tests and full reporting of outcomes
☐	☒	Estimates of effect sizes (e.g. Cohen's *d*, Pearson's *r*), indicating how they were calculated

Our web collection on statistics for biologists contains articles on many of the points above.

Software and code

Policy information about availability of computer code

Data collection	CASP for Comet Assay The Image Lab software (Bio-Rad Laboratories, Inc.) for calculate the relative band of WB.
Data analysis	no software was used

For manuscripts utilizing custom algorithms or software that are central to the research but not yet described in published literature, software must be made available to editors and reviewers. We strongly encourage code deposition in a community repository (e.g. GitHub). See the Nature Research guidelines for submitting code & software for further information.

Data

Policy information about availability of data

All manuscripts must include a data availability statement. This statement should provide the following information, where applicable:

- Accession codes, unique identifiers, or web links for publicly available datasets
- A list of figures that have associated raw data
- A description of any restrictions on data availability

The uncropped blots are shown in Supplementary Information. The Source data shown in figures are provided in the file of Supplementary Data. All other data in the published article or supplementary files are available within the manuscript files.

Field-specific reporting

Please select the one below that is the best fit for your research. If you are not sure, read the appropriate sections before making your selection.

☒ Life sciences ☐ Behavioural & social sciences ☐ Ecological, evolutionary & environmental sciences

For a reference copy of the document with all sections, see nature.com/documents/nr-reporting-summary-flat.pdf

Life sciences study design

All studies must disclose on these points even when the disclosure is negative.

Sample size	All data were presented as the means of more than six parallel tests and standard derivations.
Data exclusions	No data were excluded from the analyses.
Replication	The measures for cytological study were verified more than twice. In consideration of the animal ethics, the measures for animal study were not replicated. But the sample size were enlarged to 10 for each group.
Randomization	All the samples were allocated randomly.
Blinding	The investigators were blinded to group allocation during data collection and analysis.

Reporting for specific materials, systems and methods

We require information from authors about some types of materials, experimental systems and methods used in many studies. Here, indicate whether each material, system or method listed is relevant to your study. If you are not sure if a list item applies to your research, read the appropriate section before selecting a response.

Materials & experimental systems

n/a	Involved in the study	
☐	☒	Antibodies
☐	☒	Eukaryotic cell lines
☒	☐	Palaeontology and archaeology
☐	☒	Animals and other organisms
☒	☐	Human research participants
☒	☐	Clinical data
☒	☐	Dual use research of concern

Methods

n/a	Involved in the study	
☒	☐	ChIP-seq
☒	☐	Flow cytometry
☒	☐	MRI-based neuroimaging

Antibodies

Antibodies used	1. anti-FOLR1 SAB1410427, Sigma 2. FITC-tagged secondary antibody (A16024, Thermo
Validation	The primary antibody was obtained from Sigma. The validation statements were decleared on the webside of https://www.sigmaaldrich.com/catalog/product/sigma/sab1410427?lang=zh&region=CN.

Eukaryotic cell lines

Policy information about cell lines

Cell line source(s)	L929 cell line: Human; HeLa cell line: Human
Authentication	All the cell lines were obtained from ATCC, which are authentication by the company.
Mycoplasma contamination	All the cell lines were obtained from ATCC, which confirmed that the cells were not contaminated by Mycoplasma.
Commonly misidentified lines (See ICLAC register)	No

Animals and other organisms

Policy information about studies involving animals; ARRIVE guidelines recommended for reporting animal research

Laboratory animals	Female BALB/c nude mice with aged of 4 weeks were purchased from Shanghai SLAC Laboratory Animal Co., Ltd
Wild animals	The animal procedures were all performed at the Laboratory Animal Center of Wenzhou Medical University and following the guidelines of the Laboratory Animal Ethics Committee of Wenzhou Medical University. All mice will be euthanized and burned. The spleen, kidney, liver, lung, heart and brain will be collected.
Field-collected samples	The animal procedures were all performed at the Laboratory Animal Center of Wenzhou Medical University and following the guidelines of the Laboratory Animal Ethics Committee of Wenzhou Medical University.
Ethics oversight	The ethics oversight was approved by Laboratory Animal Ethics Committee of Wenzhou Medical University & Laboratory Animal Centre of Wenzhou Medical University.

Note that full information on the approval of the study protocol must also be provided in the manuscript.
